# The atypical subunit composition of respiratory complexes I and IV is associated with original extra structural domains in *Euglena gracilis*

**DOI:** 10.1038/s41598-018-28039-z

**Published:** 2018-06-26

**Authors:** H. V. Miranda-Astudillo, K. N. S. Yadav, L. Colina-Tenorio, F. Bouillenne, H. Degand, P. Morsomme, E. J. Boekema, P. Cardol

**Affiliations:** 1 Laboratoire de Génétique et Physiologie des microalgues, InBioS/Phytosystems, Institut de Botanique, Université de Liège, Liege, Belgium; 2 Department of Electron Microscopy, Groningen Biological Sciences and Biotechnology Institute, University of Groningen, Groningen, The Netherlands; 3 Departamento de Genética Molecular, Instituto de Fisiología Celular, Universidad Nacional Autónoma de México, Mexico, Mexico; 4 InBioS/Center for Protein Engineering, Université de Liège, Liege, Belgium; 5 Institut des Sciences de la Vie, Université Catholique de Louvain, Louvain-la-Neuve, Belgium

## Abstract

In mitochondrial oxidative phosphorylation, electron transfer from NADH or succinate to oxygen by a series of large protein complexes in the inner mitochondrial membrane (complexes I–IV) is coupled to the generation of an electrochemical proton gradient, the energy of which is utilized by complex V to generate ATP. In *Euglena gracilis*, a non-parasitic secondary green alga related to trypanosomes, these respiratory complexes totalize more than 40 Euglenozoa-specific subunits along with about 50 classical subunits described in other eukaryotes. In the present study the *Euglena* proton-pumping complexes I, III, and IV were purified from isolated mitochondria by a two-steps liquid chromatography approach. Their atypical subunit composition was further resolved and confirmed using a three-steps PAGE analysis coupled to mass spectrometry identification of peptides. The purified complexes were also observed by electron microscopy followed by single-particle analysis. Even if the overall structures of the three oxidases are similar to the structure of canonical enzymes (e.g. from mammals), additional atypical domains were observed in complexes I and IV: an extra domain located at the tip of the peripheral arm of complex I and a “helmet-like” domain on the top of the cytochrome c binding region in complex IV.

## Introduction

Mitochondria generate most of the energy in eukaryotic cells via oxidative phosphorylation (OXPHOS). This process can be separated in two parts: (i) the respiratory chain classically comprising four membrane protein complexes (complexes I to IV) and two mobile electron carriers, ubiquinone and cytochrome *c*, which together transfer electrons from reduced NADH or succinate to oxygen and establish an electrochemical proton gradient across the inner mitochondrial membrane (proton-motive force); (ii) the ATP synthase (complex V) which works like a molecular motor that utilizes the energy of the proton-motive force across the membrane to synthetize ATP from ADP and inorganic phosphate.

The NADH:ubiquinone oxidoreductase (complex I) is one of the largest membrane protein assemblies known to date and has a central role in energy production. With an apparent molecular mass of ca. 1000 kDa, it comprises 45 subunits in mammals^1^, and more than 40 components in ascomycete fungi and green plants^2^. The bacterial complex I is made up of 14 different subunits, also present in eukaryotes. This so-called “core” complex therefore represents the minimal structural form of complex I and its subunits sum up a molecular mass of approximately 530 kDa^3^. The solved structures of this complex show a characteristic “L-shape” which comprises two main arms, one membrane domain in charge of the proton pumping activity and a peripheral arm involved in the electron transfer from NADH to ubiquinone^4–6^. The eukaryotic dimeric cytochrome *bc1* complex (complex III) is composed by ten different protein subunits per monomer. Three of these subunits (cytochrome *b*, cytochrome *c1* and the Rieske iron–sulfur protein) contain redox centers and participate in electron transfer from reduced quinone to cytochrome *c*. The seven supernumerary subunits are not embedded in the membrane, they are peripherally localized at the membrane surfaces and are not directly related with the catalytic activity of the complex in the electron transport chain^7–9^. The mammalian cytochrome *c* oxidase (complex IV) is a multimeric enzyme formed by 14 subunits. The core subunits (Cox1, Cox2, and Cox3) are highly hydrophobic integral membrane proteins without substantial extramembrane domains. Their structure is highly-conserved in α-proteobacteria and eukaryotes. The remaining 11 mammalian accessory subunits are located surrounding the catalytic core^10,11^. This complex exists as a 420 kDa dimer in its crystalline state, although the fully active monomeric form can be also isolated^12–14^.

Euglenids are a group of free-living, single-celled flagellates that thrive predominantly in aquatic environments. Euglenids belong to the monophyletic group of Euglenozoa together with other heterotrophic flagellates like Symbiontida (free-living flagellates found in low-oxygen marine sediments), Diplonemea (free-living marine flagellates) and Kinetoplastea (free-living and parasitic flagellates, e.g. *Trypanosoma*)^15,16^. *Euglena gracilis*, a model organism among Euglenids, is a secondary photosynthetic unicellular eukaryote that arises from an endosymbiosis between a green alga and an ancient phagotroph euglenozoan species^17,18^. This flagellate has a remarkable adaptability to various environmental conditions. It can grow photoautotrophically, heterotrophically, and photoheterotrophically^19^ and is able to metabolize various exogenous carbon sources such as sugars, alcohols and amino acids^20^. *E. gracilis* has a mitochondrial electron transfer system constituted by the conserved OXPHOS complexes (Complexes I–V) and an alternative oxidase (AOX) sensitive to diphenylamine, salicylhydroxamic acid (SHAM), n-propylgallate and disulfiram^21–23^.

The subunit composition of the OXPHOS complexes among the Euglenozoa includes additional and atypical subunits together with some conserved classical ones^24–27^. This divergent subunit composition derives notably in an atypical structure in the dimeric ATP synthase from euglenids^28,29^. Additionally, the *Euglena* complexes I and V present higher estimated molecular masses (1.5 and 2 MDa, respectively) when compared with other eukaryotic complexes^27^. The consequences of atypical subunits or structural domains in the function of the respiratory-chain pathway is still enigmatic and only hypotheses have been formulated so far, such as a role in the response of *E. gracilis* to stress^30^, resistance to inhibitors^27,31,32^, or any specific role in the parasitic lifestyle of trypanosomes^33^. Regarding the last point, our group has shown that many additional subunits originally described in kinetoplastids^33–37^ are shared with *E. gracilis*. 19 of the 45 conventional subunits of the eukaryotic complex I are found in *E. gracilis* (ND1/4/5, NDUFS1–3/7/8, NDUFV1/2, NDUFA7/9/12/13, NDUFAB1, NDUFB7/11, NDUFC1, and CAG1/2) together with 14 trypanosomatid subunits (NDTB1/2/5/6/11/12/17/18/22/25/28/29/31/34). Amongst the 10 classical complex III subunits, eight were found in *E. gracilis* (QCR1/2/6/7/10, RIP1, COB, CYT1) together with two trypanosomatid subunits (QCRTB1/2). From the 13 mammalian CIV subunits, only seven subunits (COX1/2/3/5 A/5B/6B/8 A) could be identified in *E. gracilis* along with nine kinetoplastid subunits (COXTB1/2/4/5/6/8/10/12/16)^27^. So it is unlikely that these subunits play a specific role in the parasitic lifestyle but rather play a fundamental role in Euglenozoa.

In the present work, we studied the unusual subunit composition of proton-pumping respiratory complexes (*i.e*. complexes I, III, and IV) from *Euglena gracilis* in more detail and investigated the consequences of these atypical subunit compositions on the structural level by single-particle electron microscopy.

## Results and Discussion

### Purification of the mitochondrial complexes I, III and IV from *E. gracilis*

**Figure 1:**
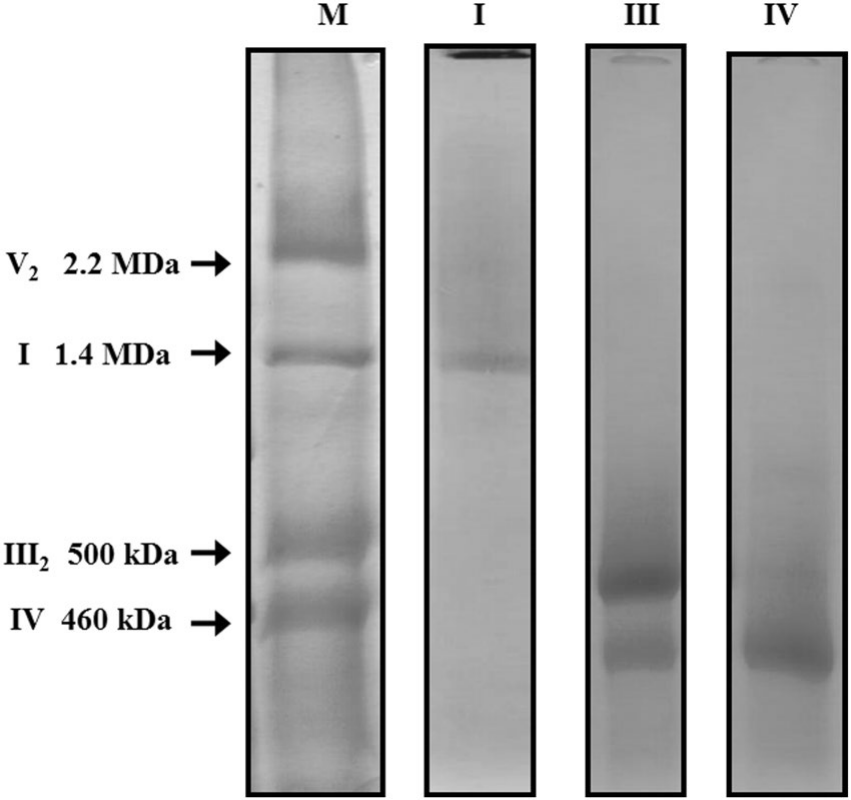
Electrophoretic patterns of purified complexes I, III and IV from *Euglena gracilis*. Isolated mitochondria were solubilized with n-dodecyl-β-D-maltoside followed by a two-step chromatographic purification (Complete gels used are shown in Suppl Information Fig. S5). (M) Total mitochondrial proteins from *Euglena gracilis*. (I) purified NADH:ubiquinone oxidoreductase (complex I) fraction, (III) enriched cytochrome bc1 complex (complex III) fraction, remaining complex IV could be observed, (IV) enriched cytochrome c oxidase (complex IV) fraction. Estimated molecular masses and identities of Coomassie brilliant blue-stained protein complexes are indicated (see Suppl. Information Fig. S1 for more details).

In a previous study we reported that the respiratory complexes of *E. gracilis* comprise many subunits which were also described in trypanosomatid respiratory complexes and, conversely, many subunits were lacking which are conserved among mammals and fungi^27^. In order to confirm this atypical subunit composition, we decided to further purify and characterize the subunit composition of complexes I, III, and IV. To this end, a purification protocol involving two chromatographic steps was developed (see Methods section for further details) to obtain enriched fractions of each of the three complexes. The fraction containing complex I was almost pure, as judged by the main 1.4 MDa complex observed after BN-PAGE analysis, while the fractions enriched in complex III (500 kDa) or complex IV (460 kDa) were slightly contaminated (Fig. 1 and Suppl. Information Fig. S1). It is to note the estimated molecular masses determined here are slightly different from the values previously reported^27^.

**Figure 2:**
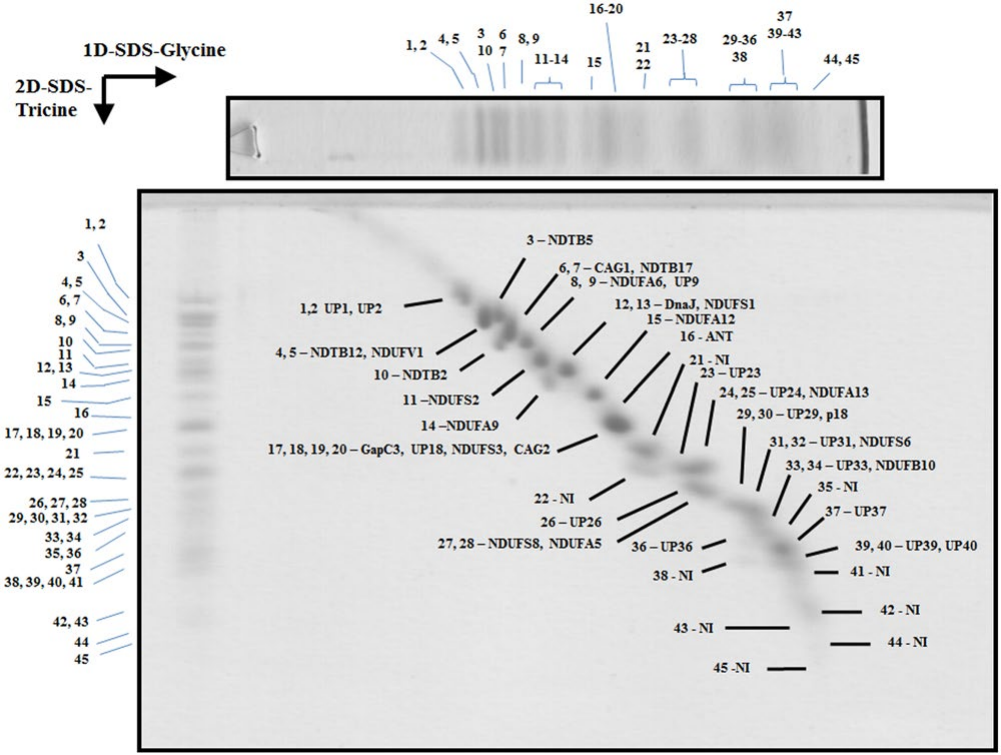
3D resolution of the polypeptides that constitute the *Euglena* mitochondrial NADH:ubiquinone oxidoreductase. Monomeric purified complex I was resolved by BN-PAGE. The band of interest was excised and resolved in a glycine-SDS-PAGE (12% acrylamide). The 2D gel was then subjected to 3D tricine-SDS-PAGE (14% acrylamide) and stained with Coomassie brilliant blue. The identified subunits are labeled. NI, not identified; UP, unnamed protein with no homolog in other species. Details about subunit identification are given in Table 1.

**Figure 3:**
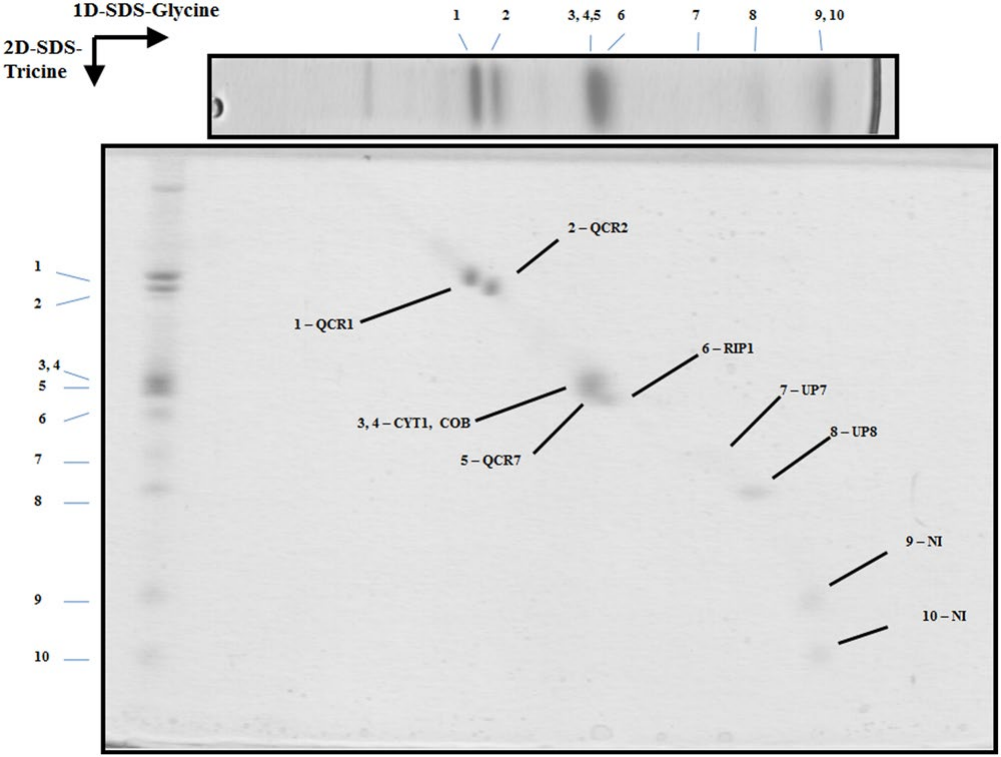
3D resolution of the polypeptides that constitute the *Euglena* mitochondrial cytochrome bc1 complex. Dimeric purified complex III was resolved by BN-PAGE. The band of interest was excised and resolved in a glycine-SDS-PAGE (12% acrylamide). The 2D gel was then subjected to 3D tricine-SDS-PAGE (14% acrylamide) and stained with Coomassie brilliant blue. The identified subunits are labeled. NI, not identified; UP, unnamed protein with no homolog in other species. Details about subunit identification are given in Table 2.

**Figure 4:**
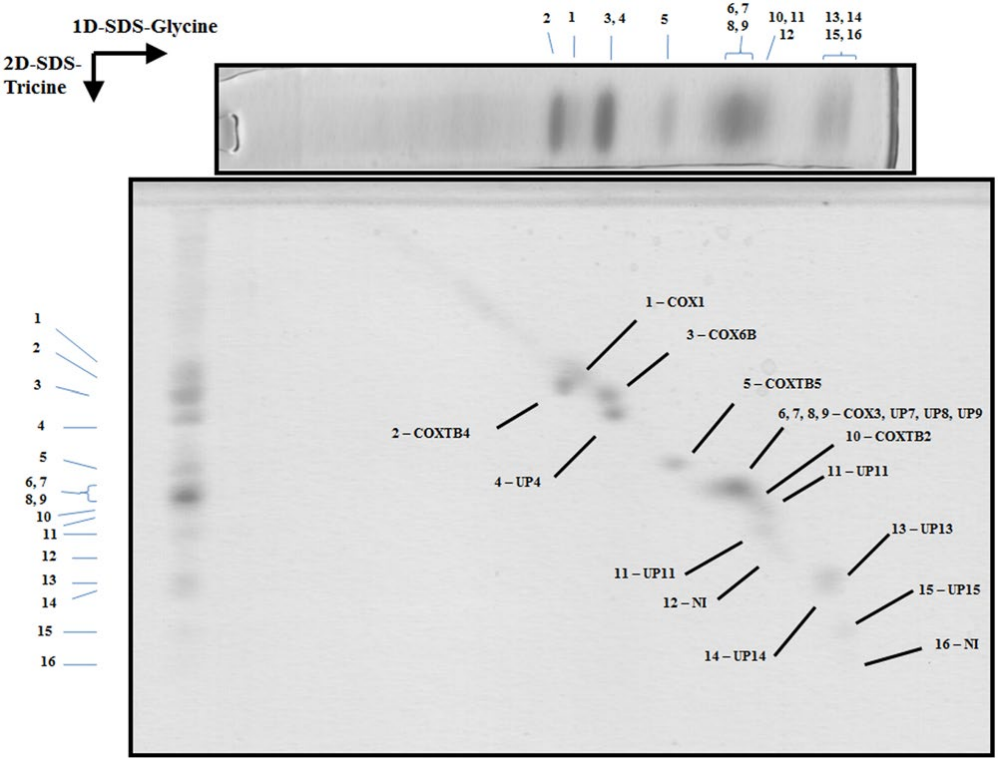
3D resolution of the polypeptides that constitute the *Euglena* mitochondrial cytochrome c oxidase. Monomeric purified complex IV was resolved by BN-PAGE. The band of interest was excised and resolved in a glycine-SDS-PAGE (12% acrylamide). The 2D gel was then subjected to 3D tricine-SDS-PAGE (14% acrylamide) and stained with Coomassie brilliant blue. The identified subunits are labeled. NI, not identified; UP, unnamed protein with no homolog in other species. Details about subunit identification are given in Table 3.

**Figure 5:**
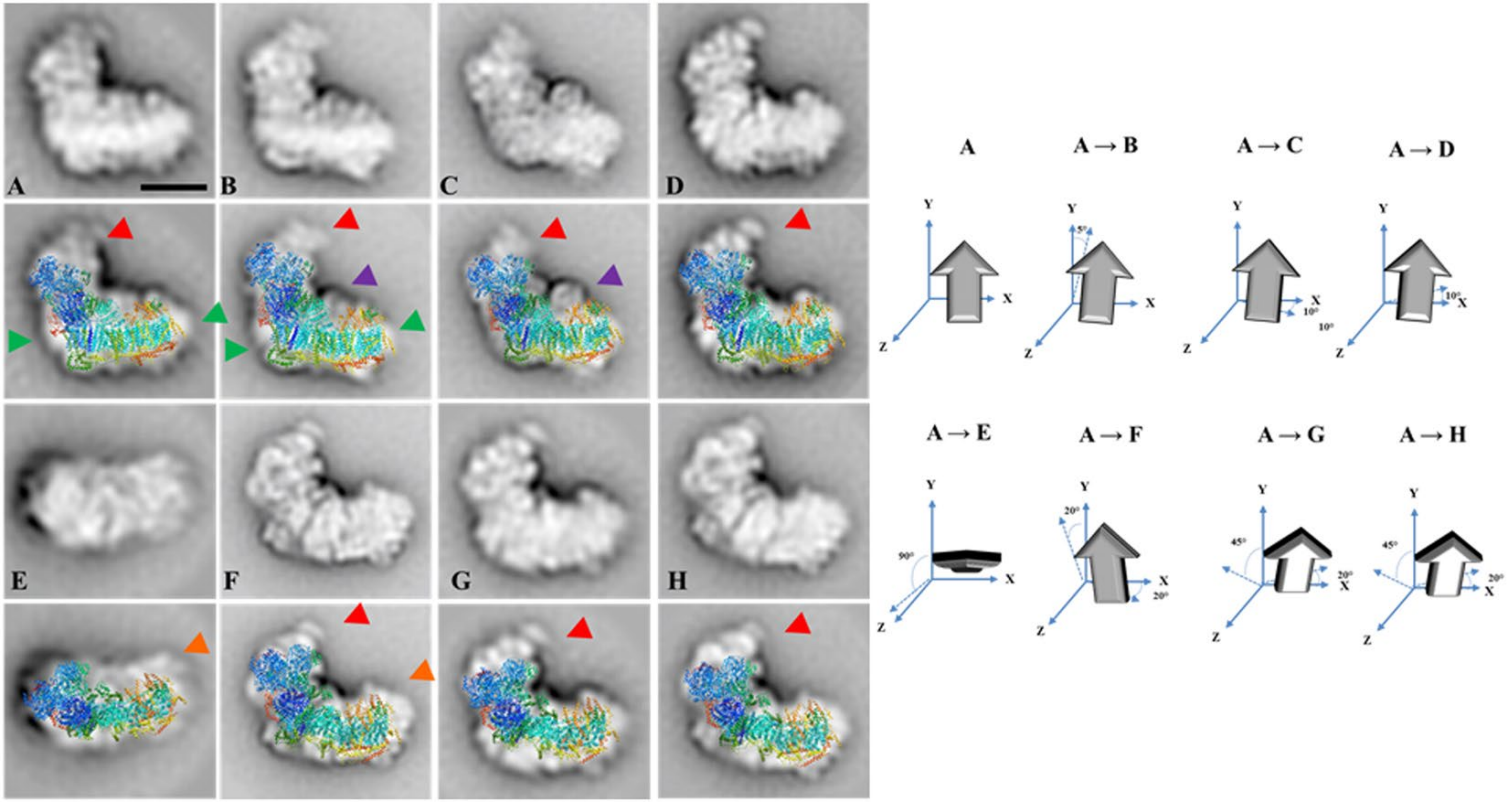
2D Projection maps of purified monomeric complex I from *E. gracilis* obtained by single particle averaging. The monomeric complex I was purified by a two-step chromatographic procedure in the presence of β-dodecyl-n-maltoside and analyzed by EM. Overlap of the bovine respiratory complex I (pdb: 5LDW^52^) over the EM images was performed. (**A**–**D**) side view projections showing the characteristic “L shape” conformation. (**E**) upper view, (**F**–**H**) almost non-tilted from a side situation. Three unusual densities are observed: (*i*) an extra domain in the tip of the peripheral arm (red arrow heads), (*ii*) matrix-exposed protuberance attached to the membrane arm at a central position (purple arrow heads) and (*iii*) an extra region located in the extreme of the hydrophobic domain (orange arrow heads). *At the right*: schematic representation of the rotation of the complex in the left panels. The approximate rotation angles over the axis are indicated. The scale bar is 10 nm.

**Figure 6:**
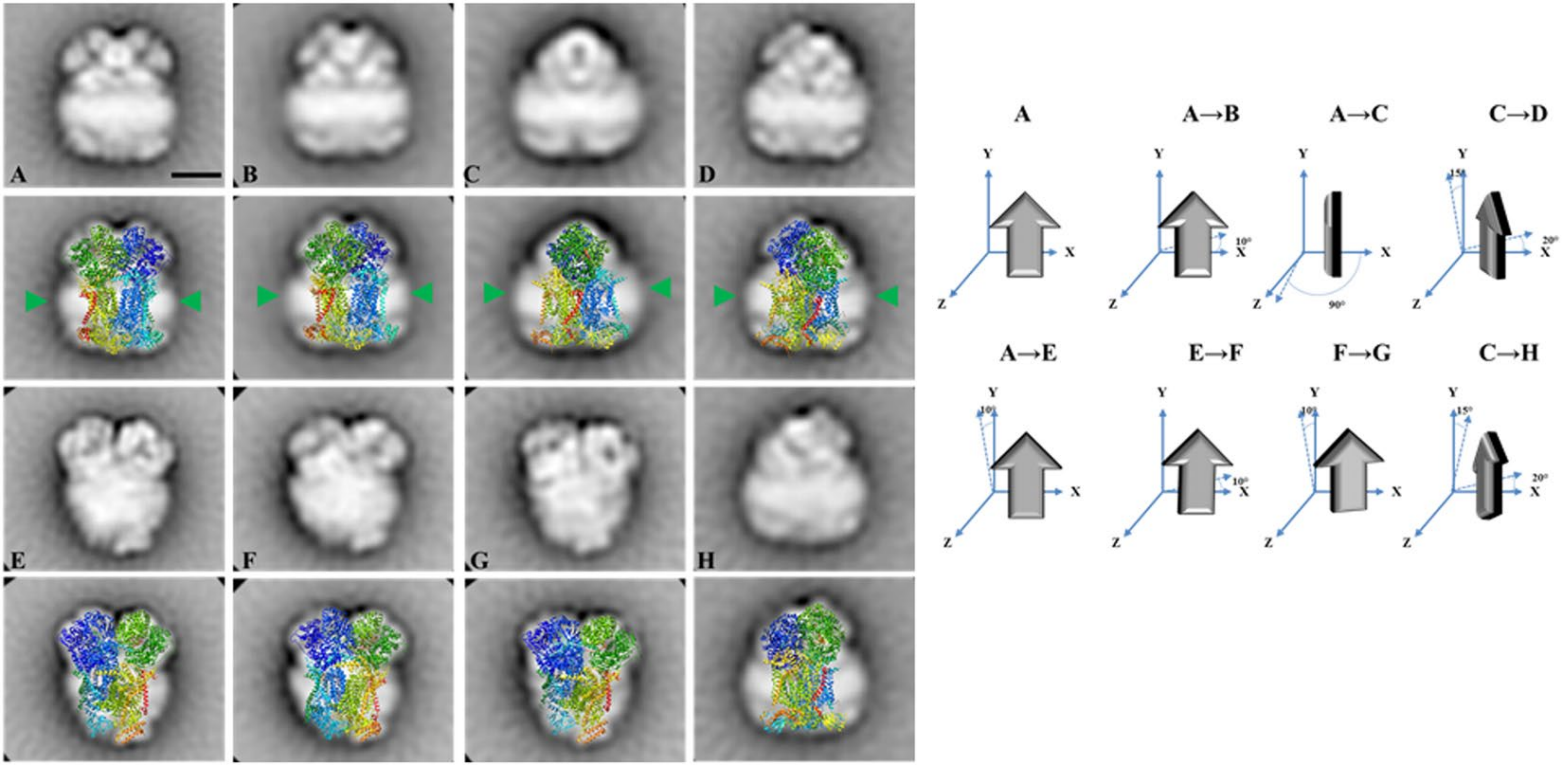
2D Projection maps of purified dimeric complex III from *E. gracilis* obtained by single particle averaging. The dimeric complex III was purified by a two-step chromatographic procedure in the presence of β-dodecyl-n-maltoside and analyzed by EM. Overlap of the chicken dimeric complex III (pdb: 4U3F^63^) over the EM images was performed. (**A**–**D**) side view projections, (**E**–**H**) slightly tilted side-view. The membrane region is indicated by the green arrow heads. *At the right*: schematic representation of the rotation of the complex in the left panels. The approximate rotation angles over the axis are indicated. The scale bar is 10 nm.

**Figure 7:**
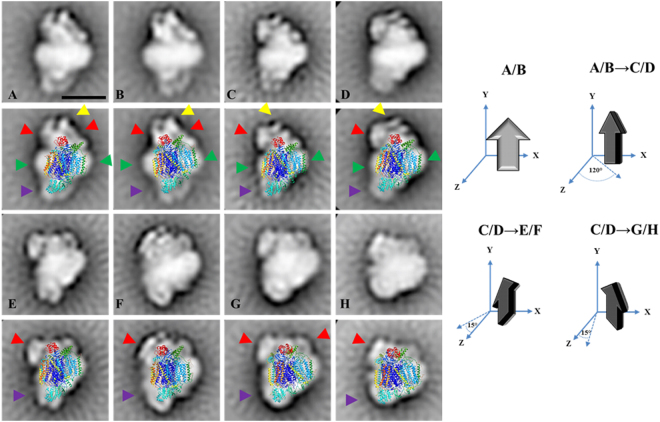
2D Projection maps of purified monomeric complex IV from *E. gracilis* obtained by single particle averaging. The monomeric complex IV was purified by a two-step chromatographic procedure in the presence of β-dodecyl-n-maltoside and analyzed by EM. Overlap of the monomeric bovine complex together with the cytochrome *c* (pdb: 5IY5^13^) over the EM images was performed. (**A** and **B**) side view projections; (**C** and **D**) ~120° rotated views from A projection along a perpendicular axis to the membrane; (**E**–**H**) slightly tilted views: the lower region is tilted by ~10–15° to the front from D projection (**E**, **F**) and the upper region is tilted by ~10–15° to the front from D projection (**G**, **H**). The membrane region is indicated by the green arrow heads, the “helmet-like” domain (see text for details) is indicated with red and yellow arrow heads, Cox5A and Cox5B positions in bovine complex IV (purple arrow heads). *At the right*: schematic representation of the rotation of the complex in the left panels. The approximate rotation angles over the axis are indicated. The scale bar is 10 nm.

The enriched complexes were applied to a 1D-BN/2D-glycine-SDS/3D-tricine-SDS gel system. The 3D gels thus obtained for each complex are shown in Figs 2–4. Compared to 2D BN-SDS PAGE analysis, the 3D gels offer a better resolution of the polypeptide components. The Coomassie-stained spots were excised from the gel and analyzed by tandem mass spectrometry (MS/MS). All the obtained sequences (Suppl. Information) were then analyzed *in silico* to search for possible homologs in protein databases (NRPS, TriTrypDB). The predicted physico-chemical properties for each subunit, such as hydrophobicity (GRAVY) and iso-electric point (IP), predicted structural features like transmembrane helices (TMH) and conserved domains (CD) were also determined by *in silico* approaches (Tables 1–3 and Suppl. Information). Finally, in order to investigate how the subunit composition of these complexes affects their overall structure, electron microscopy in combination with single particle analysis was performed. A total of *ca*. 2500–3000 raw images from each complex were recorded and analysed by electron microscopy. The particle projections from each complex were classified into several groups, out of which 8 best class averages were selected per complex (an average of 3000–4000 particles per class) (Figs 5–7). The main findings for each complex will be described and discussed in the following sections.

**Table 1:** Euglena Complex I subunits identified by mass spectrometry.

I	Name	Accession number	MW	MW (calc)	TMH (putative)	Conserved Domain
1	unknown	comp60085_c0_seq. 3	58.5	44.2	0	—^a,b^
2	unknown	comp54683_c0_seq. 1	58.5	18.1	0	—^a,b^
3	NDTB5	comp54702_c0_seq. 3	52.2	54.9	1 (C)	NDUFA9_like_SDR_a (M)^a,b^
4	NDTB12	>gnl|Egra|Contig2023_c	51.0	51.8	0	—^a,b^
5	NDUFV1	comp62912_c0_seq. 6	51.0	57.8	0	NADH dehydrogenase I subunit F (A)^a,b^
6	CAG1	comp62122_c0_seq. 3	48.5	48.7	0	LbH_gamma_CA_like (M)^a,b^
7	NDTB17	comp63840_c0_seq. 1	48.5	53.3	0	Adenylate forming domain, Class I (C)^a,b^
8	NDUFA6	comp61975_c0_seq. 5	45.7	48.7	0	—^a,b^
9	unknown	comp46408_c0_seq. 1	45.7	8.5	0	—^a,b^
10	NDTB2	comp63125_c0_seq. 1	44.8	46.4	0	2-enoyl thioester reductase (N,M)^a,b^
11	NDUFS2	comp55715_c0_seq. 3_c	41.9	48.6	1 (M)	NADH dehydrogenase subunit D; NADH:ubiquinone oxidoreductase 49 kD subunit 7 (A)^a,b^
12	DnaJ	comp60945_c0_seq. 5_c	40.0	43.6	1 (C)	DnaJ molecular chaperone homology domain(N)^a,b^
13	NDUFS1	comp61469_c0_seq. 1_c	40.0	43.8	0	NADH dehydrogenase/NADH:ubiquinone oxidoreductase 75 kD subunit (chain G) (A)^a,b^
14	NDUFA9	comp59654_c0_seq. 4	38.7	41.3	0	NADH dehydrogenase (ubiquinone) 1 alpha subcomplex, subunit 9, 39 kDa (A)^a,b^
15	NDUFA12	gnl|Egra|Contig149	35.7	23.2	0	NADH ubiquinone oxidoreductase subunit NDUFA12^a,b^
16	ADP/ATP	comp62013_c0_seq. 2_cut_gi|109788168	32.4	28.3	2(C)	Mitochondrial carrier protein x 2(N,M)^a,b^
17	GapC3	comp52123_c0_seq. 2_gi|125990644	31.0	37.1	0	Glyceraldehyde 3-phosphate dehydrogenase, C-terminal domain (C)^a,b^
18	unknown	comp58177_c0_seq. 3	31.0	32.6	3(M)	—^a,b^
19	NDUFS3	comp62960_c0_seq. 11	31.0	32.4	0	NADH dehydrogenase subunit C (M)^a,b^
20	CAG2	comp48089_c0_seq. 3_c	31.0	28.8	0	Gamma carbonic anhydrase-like (M)^a,b^
21	NI	—	27.7	—	—	—
22	NI	—	26.1	—	—	—
23	unknown	comp47716_c0_seq. 2_c	25.5	24.0	0	—^a,b^
24	unknown	comp51732_c0_seq. 5_ gi|125990596	25.1	22.6	0	—^a,b^
25	NDUFA13	comp59406_c0_seq. 3	25.1	22.7	1 (M)	GRIM-19 protein (M)^a,b^
26	unknown	comp52747_c0_seq. 1_c	22.9	22.0	2 (M)	—^a,b^
27	NDUFS8	comp54309_c0_seq. 4_c	22.4	24.3	0	NADH dehydrogenase subunit I (M)^a,b^
28	NDUFA5	comp49716_c0_seq. 2	22.4	20.8	0	ETC_C1_NDUFA5 (M)^a,b^
29	unknown	comp49825_c0_seq. 3_c	21.3	19.5	1 (C)	—^a,b^
30	p18	comp56597_c0_seq. 3	21.3	21.0	0	—^a,b^
31	unknown	comp53986_c0_seq. 1_c	20.9	18.3	0	—^a,b^
32	NDUFS6	comp55416_c0_seq. 4	20.9	17.1	0	—^a,b^
33	unknown	comp41364_c0_seq. 3	18.8	17.7	1 (C)	—^a,b^
34	NDUFB10	comp54566_c0_seq. 2	18.8	17.4	0	—^a,b^
35	NI	—	18.3			
36	unknown	comp54442_c0_seq. 1	17.5	17.2	1 (M)	—^a,b^
37	unknown	comp54200_c0_seq. 1	17.1	15.6	1 (N)	—^a,b^
38	NI	—	16.8			
39	unknown	comp51611_c0_seq. 1_c	16.3	14.9	1 (M)	—^a,b^
40	unknown	comp51117_c0_seq. 1	16.3	9.9	0	—^a,b^
41	NI	—	15.5	—	—	—
42	NI	—	11.7	—	—	—
43	NI	—	10.7	—	—	—
44	NI	—	8.7	—	—	—
45	NI	—	7.6	—	—	—
		Total	1333	1097		

NI not identified.

When accesion number ends with _c, the first Methionine was used for sequence analysis.

(N) Amino terminus, (C) Carboxi terminus, (M) Middle region, (A) Almost all sequence.

^a^CCD.

^b^DELTA-BLAST.

^c^The e-value threshold for the blast results was 10^−5^.

**Table 2:** Euglena Complex III subunits identified by mass spectrometry.

III	Name	Accession number	MW	MW (calc)	TMH (putative)	Conserved Domain^c^
1	QCR1	comp63646_c0_seq. 8_c	50.9	53.5	0	Peptidase_M16 (N), Peptidase_M16C (C)^a,b^
2	QCR2/TB1	comp60854_c0_seq. 1_c	47.9	51.1	0	Insulinase (Peptidase family M16) (N)^a,b^
3	CYT1	comp49373_c0_seq. 3_c	31.3	28.0	1 (C)	Cytochrome C1 family (A)^a,b^
4	COB	ALQ28773.1	31.3	43.9	9(A)	cytochrome b (A)^a,b^
5	QCR7	comp51517_c0_seq. 3_c	30.3	27.4	1 (N)	Ubiquinol-cytochrome C reductase complex 14kD subunit (C)^a,b^
6	RIP1	comp57996_c0_seq. 3_c	29.5	27.8	1 (M)	Rieske_cytochrome_bc1 (C)^a,b^
7	unknown	comp47102_c0_seq. 2_c	22.4	20.3	1 (C)	—^a,b^
8	unknown	comp57617_c0_seq. 1_c	18.8	17.6	0	—^a,b^
9	NI	—	14.7	—	—	—
10	NI	—	10.5	—	—	—
		Total	288	270		

NI not identified.

When accesion number ends with _c, the first Methionine was used for sequence analysis.

(N) Amino terminus, (C) Carboxi terminus, (M) Middle region, (A) Almost all sequence.

^a^CCD.

^b^DELTA-BLAST.

^c^The e-value threshold for the blast results was 10−5.

**Table 3:** Euglena Complex IV subunits identified by mass spectrometry.

IV	Name	Accession number	MW	MW (calc)	TMH (putative)	Conserved Domain
1	COX1	comp60817_c0_seq. 2_c	38.5	45.5	10(A)	Cytochrome C oxidase subunit I (A)^a,b^
2	COXTB4	comp55436_c0_seq. 3_c	36.4	35.6	0	—^a,b^
3	COX6B	comp54364_c0_seq. 4	35.1	33.0	0	—^a,b^
4	unknown	comp57506_c0_seq. 2_c	32.6	31.7	0	—^a,b^
5	COXTB5	gi|109781798_c	25.9	24.5	0	—^a,b^
6	COX3	comp54737_c0_seq. 2	22.9	27.3	3 (M, C)	Heme-copper oxidase subunit III (C)^a,b^
7	unknown	comp53543_c0_seq. 2	22.9	20.4	1 (M)	—^a,b^
8	unknown	comp51338_c0_seq. 1_c	22.9	19.8	0	—^a,b^
9	unknown	comp47102_c0_seq. 2_c	22.0	20.3	1 (C)	—^a,b^
10	COXTB2	comp54722_c0_seq. 3_c	22.0	19.7	0	—^a,b^
11	unknown	comp55710_c0_seq. 1_c	20.9	19.9	1 (M)	—^a,b^
12	NI	—	16.7	—	—	—
13	unknown	comp50120_c1_seq. 1_c	14.9	16.4	1 (N)	—^a,b^
14	unknown	comp48005_c0_seq. 1_c	13.6	13.1	1 (M)	—^a,b^
15	unknown	comp53374_c0_seq. 4_c	10.6	9.8	0	—^a,b^
16	NI	—	7.2	—	—	—
		Total	365	337		

NI not identified.

When accesion number ends with _c, the first Methionine was used for sequence analysis.

(N) Amino terminus, (C) Carboxi terminus, (M) Middle region, (A) Almost all sequence.

^a^CCD.

^b^DELTA-BLAST.

^c^The e-value threshold for the blast results was 10−5.

### The atypical subunits build the extra module in the peripheral arm of complex I

With the above described 3D gel system, a total of 31 spots were obtained for complex I (Fig. 2), compared to the 22 spots obtained in our previous study by 2D BN/SDS-PAGE analysis^27^. Ten of these spots matched for a single polypeptide, eleven matched for two peptides co-migrating, one matched for four proteins and nine spots did not lead to identification in our database. Globally, this analysis revealed the presence of at least 45 polypeptides associated to complex I, with molecular masses ranging from 7.6 to 58.5 kDa, and 36 of them were identified by MS analysis (Table 1). Six polypeptides correspond to complex I core subunits (NDUFS1/2/3/6/8, NDUFV1), eight to the α-proteobacterial ancestor (NDUFA5/6/9/12/13, NDUFB10, CAG1/2), four are Euglenozoa-specific subunits (NDTB2/5/12/17), and fourteen proteins do not present homologs and thus remain as unnamed proteins (UP). This analysis also corroborated the association of two short domains related to DNAJ and the glyceraldehyde 3-phosphate dehydrogenase (GapC3) enzymes [erroneously annotated as glycerol-3-phosphate dehydrogenase (G3PD) in our previous study^27^]. The ANT transporter and the Euglenozoa-specific ATP synthase subunit p18 were also detected bound to complex I (Table 1, subunits 16 and 30, respectively). The ANT is rather abundant in the mitochondrial inner membrane (12% of total protein)^38^, and therefore we cannot exclude contamination due to hydrophobic interactions. The euglenozoan p18 subunit has been reported to be attached to the ATP synthase complex in many euglenozoan species^25,27,28,39–42^ and, recently, its position in the complex attached externally to the F_1_ sector was determined^43^. The reason why subunit p18 is attached to complex I is unclear. The NDTB5 subunit (subunit 3, Table 1) was previously described as a paralogous of NDUFA9 subunit^27^. The euglenoid subunit presents a molecular mass higher (52.2 kDa) than the conserved subunit (38.7 kDa) (subunit 14, Table 1). This homology-pair characteristic is also present in the trypanomatid enzyme^44^, and thus suggests that euglenoids incorporated this new subunit by gene duplication.

Overall, these first results are similar to our previous study (Suppl. Information) and thus confirm the atypical subunit composition of *Euglena gracilis* complex I. The sum of the molecular masses from all the identified peptides (Table 1) is 1333 kDa, which is close to the value of 1.4 MDa estimated directly by BN-PAGE (Figs 1 and Suppl. Information S1). Additional subunits might also be present in the mature complex but have probably escaped from identification (see^27^ for a discussion about the underlying reasons). Among those subunits not identified by mass spectrometry but previously identified at genomic level for *Euglena* complex^27^, there are subunits present in canonical complex I of mammals, fungi and plants, like highly hydrophobic core mitochondrion-encoded (NAD1, NAD4, NAD6) or nucleus-encoded (NDUFV2, NDUFA7, NDUFAB1, and NDUFB7) subunits (Suppl. Information), all inherited from the alpha-proteobacterial ancestor^45^. This value of 1.4 MDa for *Euglena* complex I contrasts with the *ca*. 1 MDa value described in many other organisms including mammals^46,47^, yeasts^48,49^, green algae^50^ or flowering plants^51^, and prompted us to investigate the structure of *Euglena* complex I.

Negatively stained complex I adopts mainly three positions on the carbon support film (Fig. 5). The first four side-view projections (panels A–D) show that complex I has an “L shape” conformation, which is in agreement with the bacterial and mitochondrial complex I structures [*e.g*.^6,52^]. The membrane arm is best seen in projections A and B (green arrow heads). The other projections correspond to the upper view (panel E) and to tilted side views (panels F–H). The comparison with the structure of bovine respiratory complex I (pdb: 5LDW^52^) revealed two main differences: (i) a matrix-exposed protuberance attached to the membrane arm at a central position (visible in projections B and C, purple arrow heads) and (ii) an extra domain located at the tip of the peripheral arm (panels A–D and F–H, red arrow heads). A third putative extra region is located in the distal part of the hydrophobic domain (Panels E and F, orange arrow heads). However, we cannot exclude that this density is due to the presence of detergent micelles surrounding the membrane arm. All of these features are also highlighted upon comparison with the yeast Complex I (pdf: 4WZ7^53^) (Suppl. Information Fig. S2). The first domain (purple arrow heads) probably corresponds to the gamma-carbonic anhydrase (CAG) domain, first described in complex I from flowering plant mitochondria^54^ and later described in other organisms like the chlorophycean alga *Polytomella* sp.^55^ or the amoeboid protozoon *Acanthamoeba castellanii*^56^. Members of the CAG family have been identified in all eukaryotic lineages but not in Opisthokonts (*i.e*. mammals and fungi)^56,57^. In this respect, two members (CAG1-2) have been found by proteomic approach in *Euglena* complex I (Fig. 2 and Table 1). In contrast, the extra domain located at the tip of the peripheral arm (Fig. 5, red arrow heads) has no counterpart in the complex I structures described in representatives of other lineages. Interestingly, based on the presence of some additional subunits in trypanosomes, a fatty acid synthase (FAS) domain has been proposed as an additional complex I structural domain. Among the non-canonical subunits identified in trypanosomes^35,44^, NDTB2 and NDTB17 were also confirmed here as components of Euglena complex I. Neither NDTB2 nor NDTB17 subunits possess any putative transmembrane helices but comprise a functional domain related to fatty acid synthesis (FAS) (Table 1). The NDTB17 subunit presents an adenylate forming domain, a domain which has been associated with the fatty acid activation necessary for their subsequent metabolism (*e.g*. β-oxidation)^58,59^. The NDTB2 subunit presents a 2-enoyl thioester reductase domain. This enzymatic activity has been associated with the NADPH-dependent conversion of trans-2-enoyl acyl carrier protein/coenzyme A (ACP/CoA) to acyl-(ACP/CoA) in fatty acid synthesis^60^. NDTB2 and NDTB17 are thus good candidates to participate in a FAS domain. In trypanosomes, it was suggested that such domain is located at the interface between the membrane and matrix arms^44^, but no extra density can be distinguished in this area in *Euglena* complex I. We propose that the FAS domain is therefore located at the tip of the matrix domain in Euglenozoa (Fig. 5, and Suppl. Information Fig. S2). It should be noted that a mitochondrial small acyl carrier protein NDUFAB1/ACPM is also found associated to complex I in mammals and fungi^61^ but not in flowering plants^51^. This subunit has been found in *Euglena* at genome level (Suppl. Information). Altogether, these findings may indicate an ancient function related to fatty acid metabolism associated to complex I in Eukaryotes.

### Overall conserved subunit composition and structure of *Euglena* Complex III

*Euglena* mitochondrial complex III was resolved into 10 protein spots of molecular masses ranging between 10.5 and 50.9 kDa (Fig. 3). Six corresponded to a single polypeptide in our database, one was found to comprise two different polypeptides, and two were not identified. Six of these polypeptides corresponded to canonical complex III subunits QCR1, QCR2, QCR7, CYT1, COB, and RIP1, whereas two polypeptides of about 20 kDa do not have homologs in the databases and thus remained as unnamed proteins (UP) (Table 2 and Suppl. Information). Two additional complex III canonical subunits (QCR6/10) that were previously identified at genomic level in *Euglena* could not be identified here (Suppl. Information). No CD could be confidently identified for the two UP proteins and only one putative additional TMH is predicted for UP7 protein (e-value threshold = 10^−5^).

The reported structures for the eukaryotic dimeric complex III are composed of 10 to 11 subunits per monomer^7,62^. In *Euglena*, the sum of all the determined peptides (288 kDa per monomer, Table 2) is in good agreement with the size of a native dimeric complex (*ca*. 500 kDa, Fig. 1). The *Euglena* complex III particles classify into two principal groups on the carbon film (Fig. 6): Side-view projections (panels A–D) and slightly tilted side-view projections (panels E–H). The membrane region of the complex is evident in the side-view projections (Panels A–D green arrow heads). Panels A and B represent the side-by-side monomer placement, panel C represents the 90° rotation (one monomer in front of the other). The comparison with the structure of chicken dimeric complex III [pdb: 4U3F^63^] explains all the projections obtained and corroborates the dimeric oligomeric state of this complex. The absence of additional electronic densities from any projection concurs with the absence of extra atypical subunits. In conclusion, the overall structure of *E. gracilis* complex III is highly similar to the one reported for other species. However, the lack of QCR8 coding sequence in *Euglena* nucleotide database^27^ questions the complex III biogenesis in *Euglena*. QCR8, which has one TMH^63^, is required to form an early core subcomplex with COB and QCR7 during complex III biogenesis in *S. cerevisiae*^62,64^. The QCR7 subunit usually shields the quinone pocket of the CYT1 subunit from exposure to the aqueous environment on the matrix side^65^. Interestingly, *Euglena* QCR7 subunit presents an atypical N-terminal extension (~78 residues; Suppl. Information Fig. S3) with a putative TMH that may take the role of the missing QCR8 subunit. Another role for this QCR7 extension in *Euglena* could be to maintain the structural integrity of the quinone reduction site. Such a function has already been described for the atypical extension in the CYT1 subunit from *Rhodobacter sphaeroides bc1* complex^66^. A subunit named QCR9 was also found in *Euglena* complex III by N-terminal sequencing^67^ even though this subunit does not correspond to the canonical QCR9 subunit described in other species^27,67^. Altogether, this analysis confirmed the dimeric state of the purified complex and showed no atypical domains within the overall structure.

### Atypical subunit composition of *Euglena* complex IV is associated to the presence of an extra domain in the cytochrome c binding region

With the current 3D gel system a total of 14 spots were obtained for *Euglena* mitochondrial complex IV (Fig. 4), while it was previously resolved into 10 protein spots^27^. A single polypeptide could be identified in our database for eleven spots, one spot matched to four polypeptides of similar molecular masses and two spots remained unidentified. Three polypeptides correspond to classical complex IV subunits (COX1, COX3, and COX6B), three to Euglenozoa-specific subunits (COXTB2/4/5), six peptides do not present any similarity with other existing proteins and therefore remain as unnamed proteins (UP) (Table 3 and Suppl. Information). Here again, no CD could be confidently identified for non-classical subunits (e-value threshold = 10^−5^). Globally, our analysis allowed the identification of at least 16 polypeptides associated with *Euglena* complex IV with molecular masses ranging from 7.2 to 38.5 kDa. The stoichiometry of one subunit per monomer was proposed based on Coomassie staining^68^. Accordingly, the sum of all the associated peptides (365 kDa, Table 3) is lower than the molecular mass of 460 kDa determined for the whole complex by BN-PAGE (Fig. 1). Additional subunits might have escaped identification, such as previously identified core subunit COX2 or subunits identified at genomic level in *Euglena* like classical subunits (COX5A/5B/8 A)^27^, or even some of the originally proposed kinetoplastid complex IV subunits (COXTB1/6/8/10/12/16)^26^. This impressive shift in molecular mass when compared to the mammalian enzyme [200 kDa^46^] is therefore due to additional subunits rather than to dimerization. In this respect, classification of the 460 kDa cytochrome *c* oxidase images obtained by single-particle analysis led to four principal groups (Fig. 7). All pictures depict asymmetric structures unlikely to be dimers: side-views (panels A and B), ~120° rotated views from A projection along a perpendicular axis to the membrane (panels C and D), and slightly tilted views (panels E–H). The membrane region of the complex is highly recognizable in the non-tilted projections (panels A–D green arrow heads). The similarity with the structure of the monomeric bovine complex together with the cytochrome *c* (pdb: 5IY5^13^) was not obvious and the resulting overlays shown in Fig. 7 are subject to caution. In this regard, the opposite orientation of the complex with respect to domains located into the matrix and the intermembrane space led to more non-explained electronic densities at both side of the complex (Suppl. Information Fig. S2). Essentially, these comparisons brought two important pieces of information. They confirmed that the 460 kDa *Euglena* complex IV cannot be a dimer and is thus in a monomeric form. The overlays shown in Fig. 7 also highlighted a novel 5 nm extra density in the intermembrane space (red and yellow arrow heads). This extra density forms a “helmet-like” domain over the cyt *c* binding region. Because the projection of this extra domain varies across all the classes, we cannot exclude the possibility that in some of them this domain is not complete. For instance, the tip-like densities at the top of the domain (Fig. 7, yellow arrows) cannot be visualized on all pictures. Previous attempts to measure the *in vitro* oxidase activity using exogenous cyt *c* as electron donor revealed a specific requirement of *Euglena* complex IV for its endogenous cyt *c*^68,69^. This specificity was first explained by the atypical features found in the purified *Euglena* cyt *c*^70^ even though other species could use the *Euglena* cyt *c* as electron donor *in vitro*^69^. Our results allow us to propose that the structure of the *Euglena* complex IV possesses a specific cavity for its endogenous cytochrome *c*.

Among the supernumerary subunits described in other eukaryotic species only Cox6b, the mammalian homolog of yeast Cox12, was identified. Cox6b/Cox12 is a structural but non-essential subunit in close contact with Cox2 and Cox3 subunits in the intermembrane space region^71^. Other classical subunits, such as Cox5A and Cox5B that have been identified in the previous genomic survey, are located in the matrix side of the complex with no TMH (Fig. 7, purple arrow heads), meanwhile Cox8A, an isoform of the ubiquitous Cox8B^72^, is located in the membrane in close contact with Cox1 subunit^13^. Six additional TMHs can be observed in the high resolution structures of this complex^13,14^, they correspond to subunits Cox4I1/6A2/6 C/7A1/7B/7 C which have not been identified at genomic or proteomic level in *Euglena* (^27^ and Suppl. Information). One putative TMH is predicted in five UP sequences (UP 7, 9, 11, 13, 15). These subunits could take the place of the classical subunits inside the membrane region to maintain the overall structure of the complex. The absence of putative TMHs among the three trypanosomatid subunits (CoxTB2/4/5) and the UP4/8/15 subunits may indicate that they participate to the 150 kDa “helmet-like” domain. Altogether, our analyses indicate that the atypical subunits of the *Euglena* complex IV may be involved in the construction of an atypical structure whose specific role remains to be elucidated.

### Concluding remarks

The analysis of the subunit composition and the structure of proton-pumping respiratory oxidases (complexes I, III, and IV) of *Euglena gracilis* showed that this highly divergent organism (when compared to canonical yeast and mammal models) shares many atypical subunits described in respiratory complexes of trypanosomes^27^. While the canonical subunits and the lineage-specific subunits maintain the overall architecture of the respiratory complexes described in yeast and mammals, the lineage-specific subunits are presumably responsible for the atypical extra domains observed in the structure of monomeric complexes I and IV. Incidentally, the presence of these extra subunits/domains probably explains the shift in the molecular mass of complexes I and IV. One protein is also shared between complexes III and IV (UP7-CIII corresponds to UP9-CIV). Although we observed minor cross-contamination after the purification steps, the fact that this polypeptide is the only found in common between both complexes may suggest that it is involved in interactions that lead to the formation of the supercomplex previously observed^27^. Overall, the roles of these atypical subunits/domains in enzyme activities or supramolecular associations remain to be elucidated.

## Material and Methods

### Algal strain, growth conditions and mitochondria isolation

*Euglena gracilis* (SAG 1224-5/25) was obtained from the University of Göttingen (Sammlung von Algenkulturen, Germany). Cells were grown in the dark under orbital agitation at 25 °C. Ethanol 1% was used as carbon source. The liquid mineral Tris-minimum-phosphate medium (TMP) pH 7.0 was supplemented with a mix of vitamins (biotin 10^−7^%, B12 vitamin 10^−7^% and B1 vitamin 2 × 10^−5^% (w/v)). The cells were collected at the middle of the logarithmic phase by a 10-min centrifugation step at 7000 × *g* and stored at −70 °C until use. Mitochondria were obtained by differential centrifugation following the procedure previously described^28^ and stored at −70 °C until use. Protein concentration was determined by the Bradford method (Biorad).

### Purification of respiratory complexes

All steps were performed at 4 °C. Seventy five milligrams of mitochondrial proteins were solubilized with n-dodecyl-β-D-maltoside (DDM, 4 g detergent per g protein) in buffer A containing Tris-HCl 50 mM, amino caproic acid 50 mM, MgSO_4_ 1 mM, NaCl 50 mM, glycerol 10%, phenylmethylsulfonyl fluoride (PMSF) 1 mM, tosyl-lysyl-chloromethylketone (TLCK) 50 μg/ml (pH 8.4). The mixture was incubated with gentle stirring for 30 min, and centrifuged at 38,000 × g for 30 min. The supernatant was diluted three times in buffer A without NaCl and supplemented with DDM 0.01%. After a filtration step (0.22 μm) the sample was loaded on an anion exchange column (Mono Q HR 5/5, 1 mL) connected to an ÄKTA explorer 100 (GE Healthcare Life Sciences) equilibrated with the same buffer and washed until a base line was obtained. The column was washed with 50 mM NaCl in the same buffer (10 VC) and eluted with a 50–500 mM NaCl linear gradient (40 VC). Two milliliter fractions were collected and visualized by BN-PAGE.

The samples enriched with Complex I were pooled and concentrated with an Amicon Ultra-15 Centrifugal Filter 100 kDa (EMD Millipore) to a final volume of 500 µL and injected to a Superose 6 10/300 (GE Healthcare Life Sciences) previously equilibrated with buffer A containing 200 mM NaCl and DDM 0.01%. The elution was carried out at 0.3 mL/min, 0.5 mL fractions were collected and visualized by BN-PAGE. The samples enriched with Complex I were pooled separately and stored at −70 °C until use.

The enriched fractions with Complex III and IV from the anion exchange column were pooled separately and concentrated to a final volume of 500 µL. Each fraction was injected separately to the Superose column equilibrated with buffer A containing 300 NaCl and DDM 0.01%. The elution of each injection was carried out as described above, 0.5 mL fractions were collected and visualized by BN-PAGE, the fractions enriched with each complex were stored at −70 °C until use.

### Non-denaturating and denaturating protein electrophoresis

Each complex was resolved by BN-PAGE using a 3–10% acrylamide gradient gel at 4 °C. The first dimension band was then excised from the gel and submitted to a 2D/3D Glycine/Tricine SDS-PAGE carried out as in^73^ at room temperature. Briefly, the subunits of each complex were separated in a Glycine-SDS-PAGE (12% acrylamide) and then each 2D lane was excised and separated individually in a Tricine-SDS-PAGE (14% acrylamide).

Coomassie blue-stained protein spots were manually excised from the 3D gels and analyzed by mass spectrometry (MS) as described in^27^. Briefly, the Matrix Laser Desorption Ionization Analyses (MALDI) was performed on a 4800 MALDI time of flight (TOF/TOF) system (Applied Bisosystems). Internal digested fragments of trypsin as well as TOF/TOF Cal Mix 5 (AB Sciex) were used as internal calibration. For direct protein identification with MASCOT against our homemade database (available in https://figshare.com/s/57d2ba4ebfbb472ae3de), protein scores greater than 60 were considered as significant (P < 0.05). To determine the molecular mass of each subunit, PageRuler plus pre-stained protein ladder (Thermo Scientific) were used as size molecular markers. The molecular mass of each protein spot was calculated from its migration distance by comparing it with the migration of molecular markers (the linear regression between the logarithm of the migration distance and the molecular mass (kDa) of the molecular markers has a R^2^ of 0.989).

### *In silico* analysis of the identified subunits from the respiratory complexes

To further characterize the sequences identified by MS, each protein sequence was submitted to a tBLASTn analysis against the expressed sequence tags (EST) database from *Euglena gracilis* (taxid: 3039) available in the NCBI server. The obtained translated nucleotide sequences were manually assembled to generate the longest possible polypeptide. The resulting polypeptides were submitted to similarity searches by BLASTp against the non-redundant protein sequences database (NRPS) (http://blast.ncbi.nlm.nih.gov/Blast.cgi), and against the Kinetoplastid genomic resource database (TriTrypDB) (http://www.tritrypdb.org/tritrypdb/). The first methionine codon of each sequence was arbitrarily chosen as the putative start codon. The theoretical isoelectric point, molecular mass and grand average of hydropathicity (GRAVY) were determined using the algorithm ProtParam (http://web.expasy.org/protparam/), transmembrane helixes (TMH) were predicted using Phobius server (http://phobius.sbc.su.se/) and TMHMM Server v. 2.0 (http://www.cbs.dtu.dk/services/TMHMM-2.0/), local conserved domains (CD) were searched using the NRPS Blastp, DELTA-Blast and Conserved Domain Blast (http://www.ncbi.nlm.nih.gov/Structure/cdd/wrpsb.cgi), the e-value threshold for the *in silico* analysis results was 10^−5^, when a TMH or CD was found the topological localization inside the peptide was annotated. Clustal Omega (http://www.ebi.ac.uk/Tools/msa/clustalo/) was used for direct alignments of protein sequences.

### Visualization of isolated complexes I, III and IV by transmission electron microscopy

5 µl of each purified complex solution was absorbed onto freshly glow-discharged carbon-coated copper grids, the excess amount of sample was blotted with filter paper and subsequently stained with 2% uranyl acetate for contrast. Imaging was performed on a Tecnai T20 equipped with a LaB6 tip operating at 200 kV. The “GRACE” system for semi-automated specimen selection and data acquisition^74^ was used to record 2048 × 2048 pixel images at 133,000 × magnifications using a Gatan 4000 SP 4 K slow-scan CCD camera with a pixel size of 0.224 nm. Single particles were analyzed with the Xmipp software (including multi-reference and non-reference alignments, multivariate statistical analysis and classification, as in^75^ and RELION software^76^. The best of the class members were taken for the final class-sums.

## Electronic supplementary material

Supplemental information
