# Supplementary material for: The atypical subunit composition of respiratory complexes I and IV is associated with original extra structural domains in Euglena gracilis
Source: Sci Rep. 2018 Jun 26;8:9698. doi: 10.1038/s41598-018-28039-z (PMC6018760; doi:10.1038/s41598-018-28039-z)
Supplement: Supplementary file 1 — Supplemental information [file 41598_2018_28039_MOESM1_ESM.docx]

**The atypical subunit composition of respiratory complexes I and IV is associated with original extra structural domains in *Euglena gracilis***

Miranda-Astudillo H.V.^1^, Yadav K.N.S. ^2^, Colina-Tenorio L.^3^, Bouillenne F.^4^, Degand H.^5^, Morsomme P.^5^, Boekema E.J.^2^, Cardol P.^1*^

**Supplemental Information**


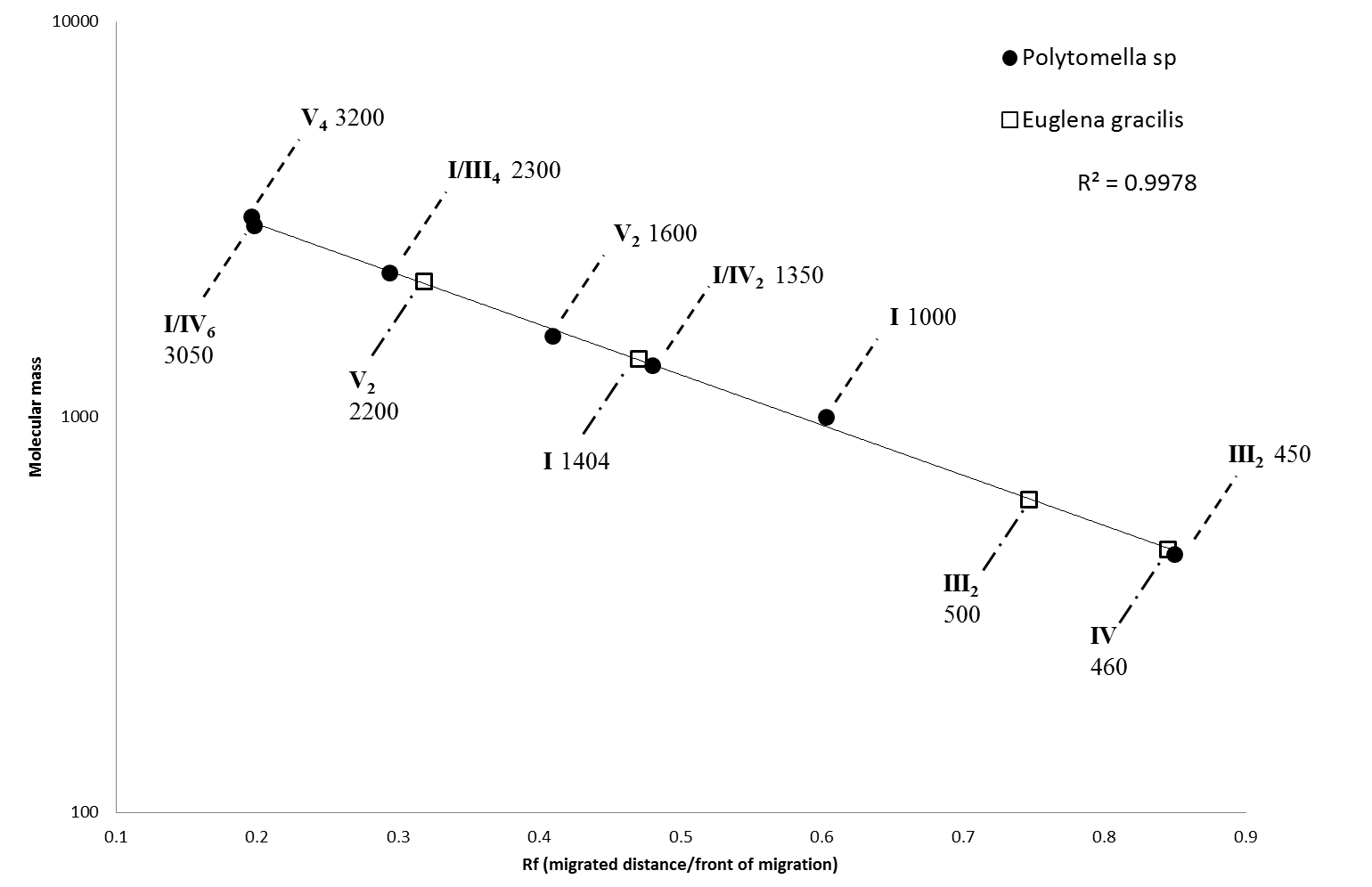


**Figure S1. Estimated molecular mass for the *Euglena respiratory* complexes.** The logarithms of the molecular masses of previously characterized chlorophycean mitochondrial complexes (●) (V_4_, V_2_, I, III_2_, I/IV_6_, I/III_4_, I/IV_2_; see ^3–8^ for details) were plotted against their migration distance in BN-PAGE. Then, the migration distances of the *Euglena* respiratory complexes (□) were interpolated and their corresponding molecular masses inferred. The stoichiometry based in EM-single particle analysis of each complex is indicated.


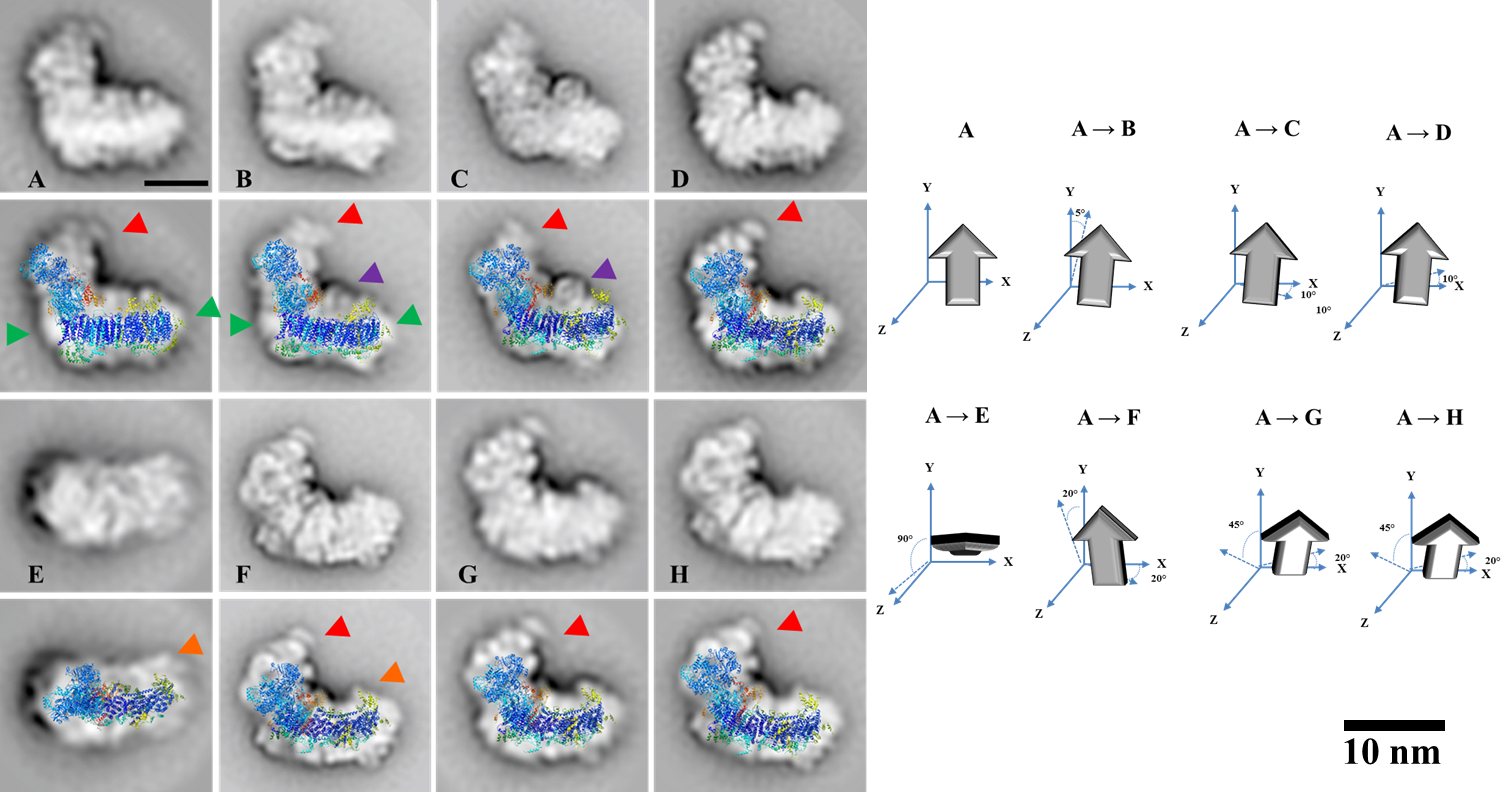


**Figure S2.** 2D Projection maps of purified monomeric complex I from *E. gracilis* obtained by single particle averaging. The monomeric complex I was purified by a two-step chromatographic procedure in the presence of β-dodecyl-n-maltoside and analyzed by EM. Overlap of yeast Complex I (pdf: 4WZ7 ^1^) over the EM images was performed. (A-D) side view projections showing the characteristic “L shape” conformation. (E) upper view, (F-H) almost non-tilted from a side situation. Three unusual densities are observed: (*i*) an extra domain in the tip of the peripheral arm (red arrow heads), (*ii*) matrix-exposed protuberance attached to the membrane arm at a central position (purple arrow heads) and (*iii*) an extra region located in the extreme of the hydrophobic domain (orange arrow heads). *At the right*: schematic representation of the rotation of the complex in the left panels. The approximate rotation angles over the axis are indicated. The scale bar is 10nm.

**Figure S3. Sequence alignment of the QCR7 from *Euglena gracilis*, Saccharomyces cerevisiae. (KZV12774.1), *Candida albicans* (XP_719189.1), *Chlamydomonas reinhardtii* (XP_001696308.1), *Volvox carteri* (EFJ52792.1) and *Kluyveromyces lactis* (CAA53617.1)**

Eg MNDAPILAKLGLSKVNTYYFQRRKHIFALPITIPNFPLAALGIFRSTDMFEERTLERYSR

Cr ------------------------------------------------------------

Vc ------------------------------------------------------------

Ca ------------------------------------------------------------

Sc ------------------------------------------------------------

Kl ------------------------------------------------------------

Eg DEREKTLGKAALLLKEAKEKGNYSELVRLDPTDPRHPYYFEHPWQSALKMDTVSLTPYQQ

Cr ----------------------MTSL------------------LKQ-----VA-LPV--

Vc ----------------------MTSL------------------LTQ-----FT-RPL--

Ca ------------------MVQSMTSVVKA------ANFILARPTLSK-----II-TPL--

Sc ------------------MPQSFTSIARI------GDYILKSPVLSK-----LC-VPV--

Kl ------------------MPQTFTSIAKI------GDYILRTPALAK-----VV-VPI--

:.: . *

Eg YLRWHCLTYRAM--HEWDRQGLLYDDLMQPKALATDPFLEEAILRLPYNQRVERERRLSR

Cr -FNSLATTYRSVVGAKLAKYGLRFDDLQDPL---KDEDVAEALRRLPPDVVVARNCRLRR

Vc -YESLARTYRAALGEKLAHYGLRFDDLQDPL---KDEDVAEALRRLPPDVVVARNCRLRR

Ca -----AQKFTAY--AGYREMGLKFNDLLLEE----TPIMQTAIKRLPSELNYSRNFRILT

Sc -----ANQFINL--AGYKKLGLKFDDLIAEE----NPIMQTALRRLPEDESYARAYRIIR

Kl -----AHQFINL--SGYRKMGLRFDDLIEEE----NELAQTALRRLPADESYARIYRIIN

. : . ** ::** *: *** : * *:

Eg AYDLALRREYLPDEDCIHPEDDVAYLHPYYHMVVDEHREQHEN-PVDVYSR----

Cr ALDLSCKHEALPKDLLEKQTPELSYLQDVLNEVRAERRERAQLGAPAPYTRIYYD

Vc ALDLSLKHEGLPADLLAKQTPELSYMQEVLAEVRAERRERAQLGAPAPYTRIYYD

Ca AHQLALSHQLLPAEKAVKPEEDDNYLIPYILEAEKEAFEKAELDNIEVKA-----

Sc AHQTELTHHLLPRNEWIKAQEDVPYLLPYILEAEAAAKEKDELDNIEVSK-----

Kl AHQLSLSHHLLPKDKWTKPEDDIPYLTPYLLEAEAFVKEKEELDNLEVAK-----

* : :. ** : : : *: . *: :


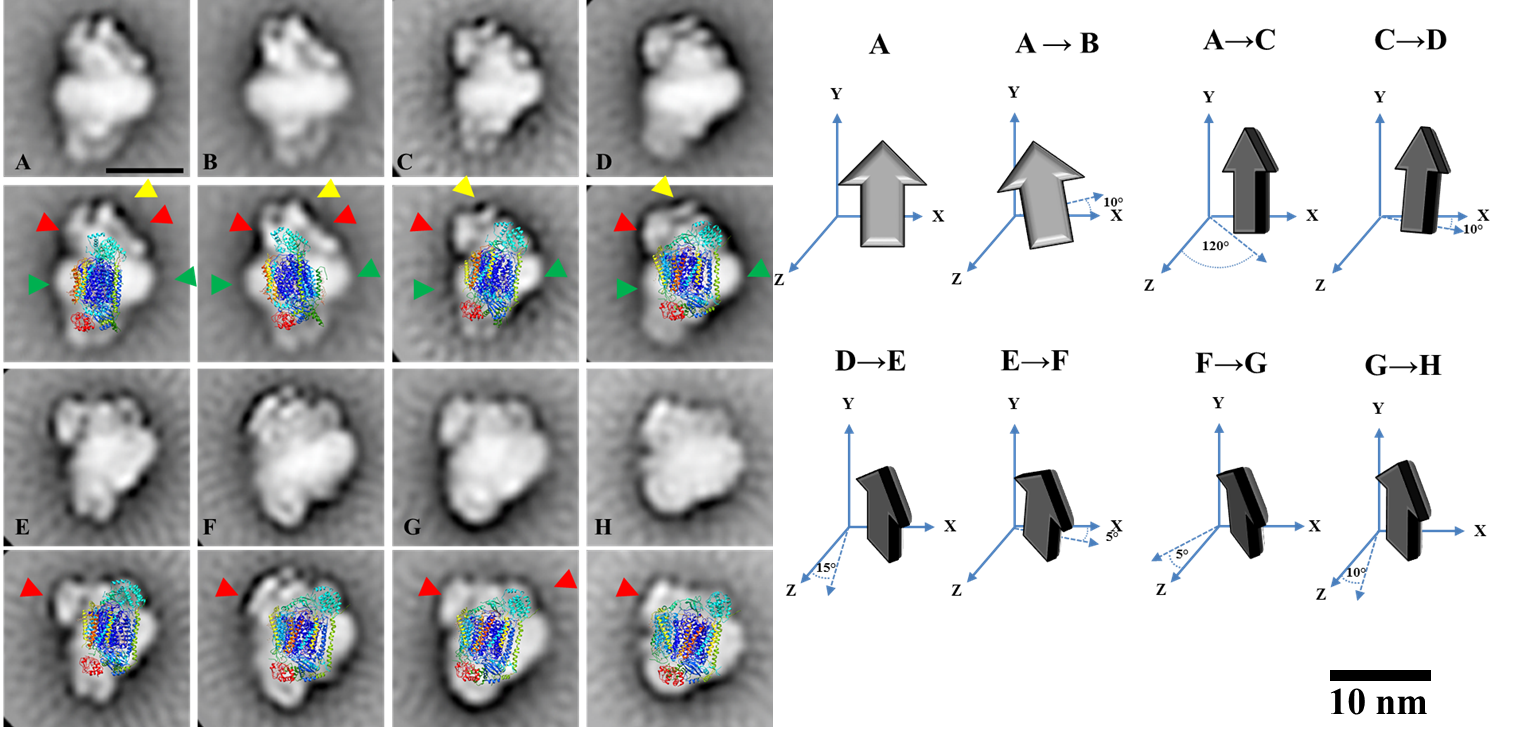


**Figure S4.** 2D Projection maps of purified monomeric complex IV from *E. gracilis* obtained by single particle averaging. The monomeric complex IV was purified by a two-step chromatographic procedure in the presence of β-dodecyl-n-maltoside and analyzed by EM. Overlap of the monomeric bovine complex together with the cytochrome *c* (pdb: 5IY5 ^2^) over the EM images was performed. (A and B) side view projections; (C and D) ~120° rotated views from A projection along a perpendicular axis to the membrane; (E-H) slightly tilted views: the lower region is tilted by ~10-15° to the front from D projection (E-F) and the upper region is tilted by ~10-15° to the front from D projection (G-H). The membrane region is indicated by the green arrow heads, extra densities are indicated with red and yellow arrow heads. *At the right*: schematic representation of the rotation of the complex in the left panels. The approximate rotation angles over the axis are indicated. The scale bar is 10 nm.


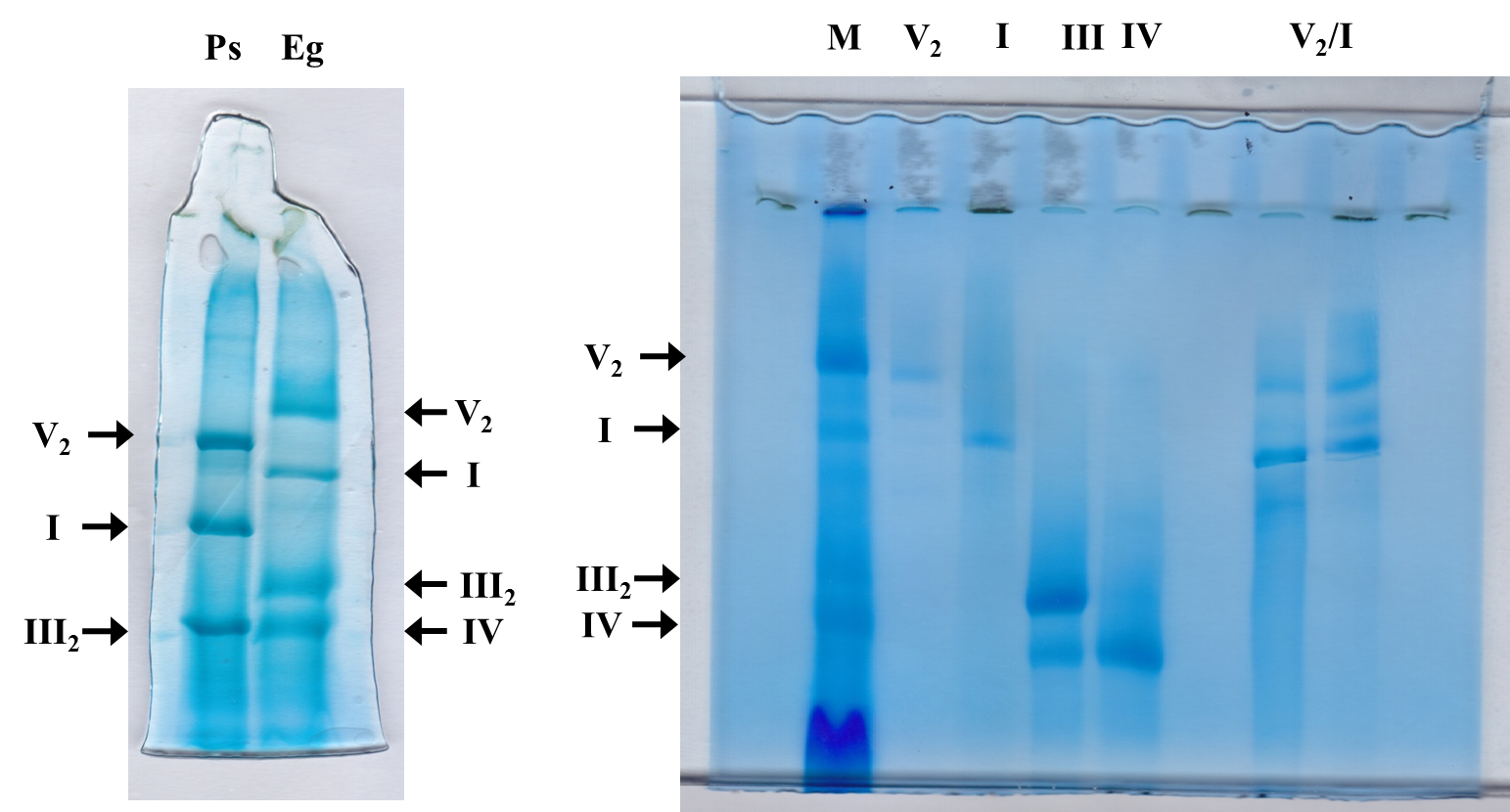


**Figure S5.** Complete gels used in Figure 1. Electrophoretic patterns of purified complexes I, III and IV from *Euglena gracilis* (Eg). The well characterized mitochondrial complexes from the chlorophycean alga Polytomella sp (Ps) we used as molecular marker.

| **I** | **Name** | **Accession number** | **MW** | **MW**  **(calc)** | **IP** | **TMH**  **(putative)** | **GRAVY** | **Conserved Domain^3^** | **Blast Hits**^3^ | **Mascot**  **Score** | **Coverage**  **%** | **Signal**  **Peptide**^¥^ |
| --- | --- | --- | --- | --- | --- | --- | --- | --- | --- | --- | --- | --- |
| 1 | unknown | comp60085_c0_seq3 | 58.5 | 44.2 | 9.3 | 0 | -0.318 | ----------^1,2^ | ------------^a,b,c^ | 508 | 40 | 20^α^  (0.6247)  44^β^  (0.9344) |
| 2 | unknown | comp54683_c0_seq1 | 58.5 | 18.1 | 9.2 | 0 | 0.338 | ----------^1,2^ | ------------^a,b,c^ | 124 | 63 | ---^αβ^ |
| 3 | NDTB5 | comp54702_c0_seq3 | 52.2 | 54.9 | 8.4 | 1 (C) | -0.209 | NDUFA9_like_SDR_a (M)^1,2^ | XP_010195242.1^a^; WP_044427727.1^b^; TRSC58_06193^c^ | 388 | 17 | ---^αβ^ |
| 4 | NDTB12 | >gnl\|Egra\|Contig2023_c | 51.0 | 51.8 | 9.3 | 0 | -0.243 | ----------^1,2^ | XP_002675541.1^a^; KFH76071.1^b^; Tc_MARK_551^c^ | 223 | 26 | ---^αβ^ |
| 5 | NDUFV1 | comp62912_c0_seq6 | 51.0 | 57.8 | 8.0 | 0 | 0.335 | NADH dehydrogenase I subunit F (A)^1,2^ | XP_010911159.1^a^; WP_020398874.1^b^; Tb11.v5.0222^c^ | 289 | 44 | ---^αβ^ |
| 6 | CAG1 | comp62122_c0_seq3 | 48.5 | 48.7 | 6.2 | 0 | -0.419 | LbH_gamma_CA_like (M)^1,2^ | XP_002670954.1^a^; WP_041963596.1^b^; Tc_MARK_8909^c^ | 795 | 40 | ---^α^  34^β^ (0.9929) |
| 7 | NDTB17 | comp63840_c0_seq1 | 48.5 | 53.3 | 8.7 | 0 | -0.182 | Adenylate forming domain, Class I (C)^1,2^ | XP_009310493.1a; WP_044747720.1b; Tgr.279.1010c | 195 | 29 | 23^α^  (0.8809)  52^β^ (0.8447) |
| 8 | NDUFA6 | comp61975_c0_seq5 | 45.7 | 48.7 | 6.1 | 0 | -0.410 | ----------^1,2^ | ------------^a,b,c^ | 652 | 57 | ---^α^  22^β^ (0.9184) |
| 9 | unknown | comp46408_c0_seq1 | 45.7 | 8.5 | 5.2 | 0 | -0.165 | ----------^1,2^ | ------------^a,b,c^ | 173 | 40 | ---^αβ^ |
| 10 | NDTB2 | comp63125_c0_seq1 | 44.8 | 46.4 | 5.8 | 0 | 0.081 | 2-enoyl thioester reductase (N,M) ^1,2^ | XP_002674454.1^a^; WP_036787526.1^b^; Tc_MARK_6161^c^ | 811 | 42 | ---^α^  13^β^ (0.8942) |
| 11 | NDUFS2 | comp55715_c0_seq3_c | 41.9 | 48.6 | 6.1 | 1 (M) | -0.079 | NADH dehydrogenase subunit D;  NADH:ubiquinone oxidoreductase 49 kD subunit 7 (A)^1,2^ | NP_066485.1a; WP_022728989.1b; ------------^c^ | 642 | 46 | 28^α^  (0.4349)  35^β^ (0.9441) |
| 12 | DnaJ | comp60945_c0_seq5_c | 40.0 | 43.6 | 8.6 | 1 (C) | -0.539 | DnaJ molecular chaperone homology domain(N) ^1,2^ | ------------^a^; BAJ64610.1^b^; TcIL3000_8_6990^c^ | 593 | 39 | 30^α^  (0.6869)  12^β^ (0.9099) |
| 13 | NDUFS1 | comp61469_c0_seq1_c | 40.0 | 43.8 | 5.5 | 0 | -0.351 | NADH dehydrogenase/NADH:ubiquinone oxidoreductase 75 kD subunit (chain G) (A) ^1,2^ | KGK35734.1^a^; EJW22228.1^b^; TCDM_05246^c^ | 117 | 18 | ---^α^  13^β^ (0.7680) |
| 14 | NDUFA9 | comp59654_c0_seq4 | 38.7 | 41.3 | 8.2 | 0 | -0.205 | NADH dehydrogenase (ubiquinone) 1 alpha subcomplex, subunit 9, 39 kDa (A)^1,2^ | WP_040187721.1^a^; WP_029042234.1^b^; Tc_MARK_4848^c^ | 317 | 52 | ---^α^  44^β^ (0.9008) |
| 15 | NDUFA12 | gnl\|Egra\|Contig149 | 35.7 | 23.2 | 10.3 | 0 | -0.759 | NADH ubiquinone oxidoreductase subunit NDUFA12^1,2^ | ------------^a,c^; XP_002752903.1^b^ | ------------ | ------------ | 34^α^  (0.9912)  17^β^ (0.7968) |
| 16 | ADP/ATP | comp62013_c0_seq2_cut_gi\|109788168 | 32.4 | 28.3 | 9.7 | 2(C) | -0.148 | Mitochondrial carrier protein x 2(N,M) ^1,2^ | ABV25601.1^a,b^; TcIL3000_10_12690^c^ | 75 | 31 | ---^αβ^ |
| 17 | GapC3 | comp52123_c0_seq2_gi\|125990644 | 31.0 | 37.1 | 8.4 | 0 | -0.648 | Glyceraldehyde 3-phosphate dehydrogenase, C-terminal domain (C)^1,2^ | AAC37245.1^a^; WP_014347265.1^b^; TCDM_02134^c^ | 119 | 13 | ---^αβ^ |
| 18 | unknown | comp58177_c0_seq3 | 31.0 | 32.6 | 9.3 | 3(M) | -0.086 | ----------^1,2^ | ------------^a,b,c^ | 84 | 29 | ---^αβ^ |
| 19 | NDUFS3 | comp62960_c0_seq11 | 31.0 | 32.4 | 6.0 | 0 | -0.512 | NADH dehydrogenase subunit C (M)^1,2^ | NP_042261.1^a^; WP_012334123.1^b^; ------------^c^ | 322 | 43 | ---^αβ^ |
| 20 | CAG2 | comp48089_c0_seq3_c | 31.0 | 28.8 | 7.8 | 0 | -0.122 | Gamma carbonic anhydrase-like (M) ^1,2^ | XP_002673591.1^a^; XP_002673591.1^b^; TCDM_08583^c^ | 259 | 43 | ---^α^  19^β^ (0.3334) |
| 21 | NI | -------- | 27.7 | --- | --- | --- | --- | --------- | --------- | --- | --- | --- |
| 22 | NI | --------- | 26.1 | --- | --- | --- | --- | --------- | --------- | --- | --- | --- |
| 23 | unknown | comp47716_c0_seq2_c | 25.5 | 24.0 | 9.5 | 0 | -0.426 | ----------^1,2^ | ------------^a,b,c^ | 231 | 46 | ---^α^  22^β^ (0.3197) |
| 24 | unknown | comp51732_c0_seq5_ gi\|125990596 | 25.1 | 22.6 | 10.7 | 0 | -0.828 | ----------^1,2^ | ------------^a,b,c^ | 84 | 30 | 23^α^  (0.9257)  26^β^ (0.9730) |
| 25 | NDUFA13 | comp59406_c0_seq3 | 25.1 | 22.7 | 9.0 | 1 (M) | -0.611 | GRIM-19 protein (M) ^1,2^ | EPY31187.1^a^; ADO28292.1^b^; Tb427tmp.01.0640^c^ | 330 | 49 | ---^αβ^ |
| 26 | unknown | comp52747_c0_seq1_c | 22.9 | 22.0 | 8.9 | 2 (M) | -0.569 | ----------^1,2^ | ------------^a,b,c^ | 224 | 22 | ---^αβ^ |
| 27 | NDUFS8 | comp54309_c0_seq4_c | 22.4 | 24.3 | 6.2 | 0 | -0.315 | NADH dehydrogenase subunit I (M)^1,2^ | WP_011271560.1^a^; KHG28214.1^b^; ------------^c^ | 102 | 48 | ---^α^  19^β^ (0.5417) |
| 28 | NDUFA5 | comp49716_c0_seq2 | 22.4 | 20.8 | 5.8 | 0 | -0.460 | ETC_C1_NDUFA5 (M)^1,2^ | CBK21264.2^a^; EPS60061.1^b^; TRSC58_00415^c^ | 610 | 59 | ---^α^  19^β^ (0.9912) |
| 29 | unknown | comp49825_c0_seq3_c | 21.3 | 19.5 | 9.7 | 1 (C) | -0.429 | ----------^1,2^ | ------------^a,b,c^ | 143 | 45 | ---^α^  17^β^ (0.6780) |
| 30 | p18 | comp56597_c0_seq3 | 21.3 | 21.0 | 8.4 | 0 | 0.205 | ----------^1,2^ | EPY41275.1^a^; CDX69249.1^b^; TcCLB.510821.60^c^ | 85 | 33 | ---^α^  16^β^ (0.9847) |
| 31 | unknown | comp53986_c0_seq1_c | 20.9 | 18.3 | 9.3 | 0 | -0.659 | ----------^1,2^ | ------------^a,b,c^ | 169 | 60 | ---^αβ^ |
| 32 | NDUFS6 | comp55416_c0_seq4 | 20.9 | 17.1 | 9.8 | 0 | -0.699 | ----------^1,2^ | XP_002111533.1^b^; ------------^a,c^ | 78 | 13 | ---^α^  29^β^ (0.8317) |
| 33 | unknown | comp41364_c0_seq3 | 18.8 | 17.7 | 9.6 | 1 (C) | -0.732 | ----------^1,2^ | ------------^a,b,c^ | 165 | 16 | ---^αβ^ |
| 34 | NDUFB10 | comp54566_c0_seq2 | 18.8 | 17.4 | 7.0 | 0 | -0.862 | ----------^1,2^ | ------------^a,b,c^ | 125 | 65 | ---^αβ^ |
| 35 | NI | ---------- | 18.3 |  |  |  |  |  |  |  |  |  |
| 36 | unknown | comp54442_c0_seq1 | 17.5 | 17.2 | 7.1 | 1 (M) | -0.603 | ----------^1,2^ | ------------^a,b,c^ | 191 | 29 | ---^αβ^ |
| 37 | unknown | comp54200_c0_seq1 | 17.1 | 15.6 | 8.6 | 1 (N) | -0.467 | ----------^1,2^ | ------------^a,b,c^ | 85 | 30 | ---^αβ^ |
| 38 | NI | ---------- | 16.8 |  |  |  |  |  |  |  |  |  |
| 39 | unknown | comp51611_c0_seq1_c | 16.3 | 14.9 | 9.7 | 1 (M) | -0.479 | ----------^1,2^ | ------------^a,b,c^ | 181 | 40 | ---^αβ^ |
| 40 | unknown | comp51117_c0_seq1 | 16.3 | 9.9 | 8.5 | 0 | -0.807 | ----------^1,2^ | ------------^a,b,c^ | 234 | 40 | ---^αβ^ |
| 41 | NI | ---------- | 15.5 | --- | --- | --- | --- | --------- | --------- | --- | --- | --- |
| 42 | NI | ---------- | 11.7 | --- | --- | --- | --- | --------- | --------- | --- | --- | --- |
| 43 | NI | ---------- | 10.7 | --- | --- | --- | --- | --------- | --------- | --- | --- | --- |
| 44 | NI | ---------- | 8.7 | --- | --- | --- | --- | --------- | --------- | --- | --- | --- |
| 45 | NI | ---------- | 7.6 | --- | --- | --- | --- | --------- | --------- | --- | --- | --- |
|  | | Total | 1333 | 1097 |  | | | | | | | |

| **III** | **Name** | **Accession number** | **MW** | **MW**  **(calc)** | **IP** | **TMH**  **(putative)** | **GRAVY** | **Conserved Domain^3^** | **Blast Hits^3^** | **Mascot**  **Score** | **Coverage**  **%** | **Signal**  **Peptide^¥^** |
| --- | --- | --- | --- | --- | --- | --- | --- | --- | --- | --- | --- | --- |
| 1 | QCR1 | comp63646_c0_seq8_c | 50.9 | 53.5 | 5.8 | 0 | -0.363 | Peptidase_M16 (N), Peptidase_M16C (C) ^1,2^ | P43264.1^a,b^; TRSC58_03965^c^ | 489 | 49 | ---^α^  39 ^β^  (0.9744) |
| 2 | QCR2/TB1 | comp60854_c0_seq1_c | 47.9 | 51.1 | 8.3 | 0 | -0.059 | Insulinase (Peptidase family M16) (N)^1,2^ | P43265.1^a,b^; Tgr.89.1110^c^ | 974 | 54 | ---^α^  25^β^  (0.9801) |
| 3 | CYT1 | comp49373_c0_seq3_c | 31.3 | 28.0 | 7.0 | 1 (C) | -0.531 | Cytochrome C1 family (A) ^1,2^ | P20114.1^a^; WP_016926090.1^b^; TcIL3000_8_1840^c^ | 77 | 33 | ---^α,β^ |
| 4 | COB | ALQ28773.1 | 31.3 | 43.9 | 8.9 | 9(A) | 0.881 | cytochrome b (A)^1,2^ | EPS72083.1^a,b^ TRSC58_07718^c^ | 36 | 8 | ---^α^  23^β^  (0.6214) |
| 5 | QCR7 | comp51517_c0_seq3_c | 30.3 | 27.4 | 6.1 | 1 (N) | -0.746 | Ubiquinol-cytochrome C reductase complex 14kD subunit (C) ^1,2^ | XP_002672796.1^a^; XP_452231.1^b^; TvY486_1004260^c^ | 552 | 65 | ---^α,β^ |
| 6 | RIP1 | comp57996_c0_seq3_c | 29.5 | 27.8 | 8.3 | 1 (M) | -0.256 | Rieske_cytochrome_bc1 (C) ^1,2^ | BAN21413.1^a^; WP_034994199.1^b^; Tc_MARK_4556^c^ | 212 | 39 | ---^α^  38 ^β^  (0.7574) |
| 7 | unknown | comp47102_c0_seq2_c | 22.4 | 20.3 | 9.9 | 1 (C) | -0.503 | ----------^1,2^ | ------------^a,b,c^; | 87 | 32 | ---^α,β^ |
| 8 | unknown | comp57617_c0_seq1_c | 18.8 | 17.6 | 10.1 | 0 | -0.254 | ----------^1,2^ | ------------^a,b,c^; | 401 | 69 | ---^α,β^ |
| 9 | NI | --------- | 14.7 | --- | --- | --- | --- | --------- | --------- | --- | --- | --- |
| 10 | NI | --------- | 10.5 | --- | --- | --- | --- | --------- | --------- | --- | --- | --- |
|  | | Total | 288 | 270 |  | | | | | | | |

| **IV** | **Name** | **Accession number** | **MW** | **MW**  **(calc)** | **IP** | **TMH**  **(putative)** | **GRAVY** | **Conserved Domain^3^** | **Blast Hits**^3^ | **Mascot**  **Score** | **Coverage**  **%** | **Signal**  **Peptide**^¥^ |
| --- | --- | --- | --- | --- | --- | --- | --- | --- | --- | --- | --- | --- |
| 1 | COX1 | comp60817_c0_seq2_c | 38.5 | 45.5 | 8.3 | 10(A) | 0.756 | Cytochrome C oxidase subunit I (A) ^1,2^ | AAB03570.1^a^; AAV68252.1^b^; TRSC58_07720^c^ | 47 | 6 | ---^α^  103^β^  (0.1790) |
| 2 | COXTB4 | comp55436_c0_seq3_c | 36.4 | 35.6 | 5.3 | 0 | -0.435 | ----------^1,2^ | AAF13293.1^a^; XP_003873171.1^b^; TCDM_05015^c^ | 794 | 58 | ---^α^  19^β^  (0.9242) |
| 3 | COX6B | comp54364_c0_seq4 | 35.1 | 33.0 | 5.6 | 0 | -0.684 | ----------^1,2^ | ------------^a,b,c^ | 599 | 43 | ---^α,β^ |
| 4 | unknown | comp57506_c0_seq2_c | 32.6 | 31.7 | 6.5 | 0 | -0.431 | ----------^1,2^ | ------------^a,b,c^ | 956 | 59 | ---^α^  24^β^  (0.5641) |
| 5 | COXTB5 | gi\|109781798_c | 25.9 | 24.5 | 6.1 | 0 | 0.135 | ----------^1,2^ | XP_803621.1^a^; XP_011776354.1^b^; Tb427tmp.160.1820^c^ | 197 | 22 | ---^α^  13^β^  (0.7981) |
| 6 | COX3 | comp54737_c0_seq2 | 22.9 | 27.3 | 6.8 | 3 (M, C) | 0.363 | Heme-copper oxidase subunit III (C)^1,2^ | WP_036357778.1a; YP_001122867.1b; ------------^c^ | 137 | 18 | ---^α^  22^β^  (0.8246) |
| 7 | unknown | comp53543_c0_seq2 | 22.9 | 20.4 | 9.4 | 1 (M) | -0.489 | ----------^1,2^ | ------------^a,b,c^ | 204 | 49 | ---^α,β^ |
| 8 | unknown | comp51338_c0_seq1_c | 22.9 | 19.8 | 6.2 | 0 | -0.722 | ----------^1,2^ | ------------^a,b,c^ | 203 | 27 | ---^α^  28^β^  (0.4229) |
| 9 | unknown | comp47102_c0_seq2_c | 22.0 | 20.3 | 9.9 | 1 (C) | -0.503 | ----------^1,2^ | ------------^a,b,c^ | 355 | 49 | ---^α,β^ |
| 10 | COXTB2 | comp54722_c0_seq3_c | 22.0 | 19.7 | 5.6 | 0 | -0.298 | ----------^1, 2^ | KNH05523.1^a^; WP_049757201.1^b^; Tb427.10.4880^c^ | 40 | 28 | 21^α^  (0.4919)  14^β^  (0.9158) |
| 11 | unknown | comp55710_c0_seq1_c | 20.9 | 19.9 | 6.1 | 1 (M) | -0.309 | ----------^1,2^ | ------------^a,b,c^ | 462 | 45 | ---^α^  30^β^  (0.8889) |
| 12 | NI | ----------- | 16.7 | --- | --- | --- | --- | ---------- | ---------- | --- | --- | --- |
| 13 | unknown | comp50120_c1_seq1_c | 14.9 | 16.4 | 9.4 | 1 (N) | -0.327 | ----------^1,2^ | ------------^a,b,c^ | 218 | 27 | ---^α,β^ |
| 14 | unknown | comp48005_c0_seq1_c | 13.6 | 13.1 | 9.7 | 1 (M) | -0.267 | ----------^1,2^ | ------------^a,b,c^ | 130 | 56 | ---^α^  82^β^  (0.9061) |
| 15 | unknown | comp53374_c0_seq4_c | 10.6 | 9.8 | 10.3 | 0 | -0.487 | ----------^1,2^ | ------------^a,b,c^ | 274 | 58 | ---^α^  72^β^  (0.5104) |
| 16 | NI | ---------- | 7.2 | --- | --- | --- | --- | ---------- | ---------- | --- | --- | --- |
|  | | Total | 365 | 337 |  | | | | | | | |

NI not identified.

_c start from the first Methionine.

(N) Amino terminus, (C) Carboxi terminus, (M) Middle region, (A) Almost all sequence.

^α^ Phobius; Cleavage site (Probability of the peptide signal).

^β^ MitoProt; Cleavage site (Probability of export to mitochondria).

^a^ Blastp.

^b^ DELTA-BLAST.

^c^ BLAST TriTrypDB.

^1^ CCD.

^2^ DELTA-BLAST.

^3^ The e-value threshold for the blast results was 10^−5^.

^¥^ % Probability (Region).

| **Complex I** | | **Complex III** | | **Complex IV** | |
| --- | --- | --- | --- | --- | --- |
| **Perez et al., 2014** | **Present work** | **Perez et al., 2014** | **Present work** | **Perez et al., 2014** | **Present work** |
| **Core** | | **Classical** | | **mammalian CIV** | |
| ND1* | - | QCR1 | QCR1 (Subunit 1) | COX1 | COX1 (Subunit 1) |
| ND4* | - | QCR2 | QCR1 (Subunit 2) | COX2 | - |
| ND5* | - | RIP1 | RIP1 (Subunit 6) | COX3 | COX3 (Subunit 6) |
| NDUFS1 | NDUFS1 (Subunit 13) | COB | COB (Subunit 4) | COX5A* | - |
| NDUFS2 | NDUFS2 (Subunit 11) | CYT1 | CYT1 (Subunit 3) | COX5B* | - |
| NDUFS3 | NDUFS3 (Subunit 19) | QCR6* | - | COX6B | COX6B (Subunit 3) |
| - | NDUFS6 (Subunit 32) | QCR7 | QCR7 (Subunit 5) | COX8A* | - |
| NDUFS7 | - | QCR10* | - | **kinetoplastid-specific subunits** | |
| NDUFS8 | NDUFS8 (Subunit 27) | **kinetoplastid-specific** | | COXTB1* | - |
| NDUFV1 | NDUFV1 (Subunit 5) | QCRTB1 | QCRTB1 (Subunit 2) | COXTB2 | COXTB2 (Subunit 10) |
| NDUFV2* | - | QCRTB2* | - | COXTB4 | COXTB4 (Subunit 2) |
| **α-proteobacterial ancestor** | | **Others** | | COXTB5 | COXTB5 (Subunit 5) |
| NDUFA7* | - | UP6 | UP8 | COXTB6* | - |
| - | NDUFA6 (Subunit 8) | UP7 | - | COXTB8* | - |
| - | NDUFA5 (Subunit 28) | - | UP7 | COXTB10* | - |
| NDUFA8 | - |  | | COXTB12* | - |
| NDUFA9 | NDUFA9 (Subunit 14) |  |  | COXTB16* | - |
| NDUFA12 | NDUFA12 (Subunit 15) |  |  | **Others** | |
| NDUFA13 | NDUFA13 (Subunit 25) |  |  | UP3 | UP4 |
| NDUFAB1* | - |  |  | UP5 | UP9, UP8 |
| NDUFB7* | - |  |  | UP6 | UP7 |
| - | NDUFB10 (Subunit 34) |  |  | UP7 | UP11 |
| NDUFB11* | - |  |  | UP8 | UP13 |
| NDUFC1* | - |  |  | UP10 | - |
| CAG9 (CAG1) | CAG1 (Subunit 6) |  |  | - | UP14 |
| CAG9 (CAG2) | CAG2 (Subunit 20) |  |  | - | UP15 |
| **kinetoplastid-specific** | |  |  |  | |
| NDTB1* | - |  |  |  |  |
| NDTB2 | NDTB2 (Subunit 10) |  |  |  |  |
| NDTB5 | NDTB5 (Subunit 3) |  |  |  |  |
| NDTB6* | - |  |  |  |  |
| NDTB11* | - |  |  |  |  |
| NDTB12 | NDTB12 (Subunit 4) |  |  |  |  |
| NDTB17 | NDTB17 (Subunit 7) |  |  |  |  |
| NDTB18* | - |  |  |  |  |
| NDTB22* | - |  |  |  |  |
| NDTB25* | - |  |  |  |  |
| NDTB28* | - |  |  |  |  |
| NDTB29 | - |  |  |  |  |
| NDTB31* | - |  |  |  |  |
| NDTB34* | - |  |  |  |  |
| **Others** | |  |  |  |  |
| DnaJ | DnaJ (Subunit 12) |  |  |  |  |
| G3PD | Gap3C (Subunit 17) |  |  |  |  |
| - | ANT (Subunit 16) |  |  |  |  |
| - | p18 (Subunit 30) |  |  |  |  |
| UP1 | UP 1 |  |  |  |  |
| UP13 | UP18 |  |  |  |  |
| UP14 | - |  |  |  |  |
| UP17 | UP31 |  |  |  |  |
| UP18 | UP33 |  |  |  |  |
| UP19 | - |  |  |  |  |
| UP22 | UP39 |  |  |  |  |
| - | UP2 |  |  |  |  |
| - | UP9 |  |  |  |  |
| - | UP23 |  |  |  |  |
| - | UP24 |  |  |  |  |
| - | UP26 |  |  |  |  |
| - | UP29 |  |  |  |  |
| - | UP36 |  |  |  |  |
| - | UP37 |  |  |  |  |
| - | UP40 |  |  |  |  |

- Not identified; * identified at genomic level

**Sequences Complex I**

>comp60085_c0_seq3 [unknown]

LFRSFAFVTLPPNPMASGSEVLRQFLTIRKNSYKYAPAFQRLHALVNGANSAAKLRARHQKRLGINVVLGEKSDLGLCQLADTLADRLKLADLGVSARPAKSPAVYYGHLAAQQHRYAVPSELKYTESSYSSRNVYIWLWTDVQQEAPDLHTQIFTGPTSNCNVYSFGHVHNARAGVKPVGGMEEFVGWLEGRTNLFSRTPKLETRLSNVYVLYSDNFLEMFPTNYGDIFKKIEELLGDQTFVSFSYLSRHPVSYNAVQTYAFPPVTQLLKRNDQYRLNVLTNVQRQDYSENESRGRFTARLMCHSTLLRADQPMNELVIAQKTPAEDNAALAYIDKFGDYKSAINSIFISEFSDKLQLMHPHQLLTYAFALLAWPRALARLLPRSEER

>comp54683_c0_seq1 [unknown]

RLLPLTSIPKADEEKTFKATHSQFLERLIRDFDNDPTRLSLIHALSLGRPALVEDLRLRLWPYTVVPGTAFNVVKAKALLQRLNATPEYSPDGPYYEFQTPAAPVPSAAPTPAPQRVALKSDSIFAIDCEFVRHSMPLRGHINEVNRKQHLSWCKLAPESK

>comp54702_c0_seq3 [NDTB5]

MARPPAEVTMLIKNAGDLLTKVKLENPPTRLLLDPKTIKLATQDPTVKGKVKDLMLKGVKVEPSTAARVEHTFIPAPKQTENQYSKPLLGYRLRELRTKVLSNEVYSTPRPRPLRGVVATVFGGNGFLGNQVVAQLAQYGATVICPTRINNEEHPVVMNTRDFRQIKSLGDQGQVFPVVYNPTVFDEVAQCVERSQVVFNCIGGFYPAMNQSQSFGPEALFANLPRNIARACAMKGVQRLVHTSHINADVSSPIPFFKYKALGEEAVLDEFPNGIIIRPADIFGDRDNFTTLMVNLLKGSNWPIMSTNTYLLEGNEYVECQPVWVVDVARAMVRAAMREYTFGQTYQLPGPDRYKLIEVMRYIEAITQLQPSHVRVYSPLEAQLRFDRPGGENHRSWIDLHLRENVVPKPGVKTWQDLEIDNSILTKMENITGDWMSKAPYRDMPTGFDEELTDLSLPRVWGDYDKKLIAFPAVSAVAAVLYALAILFP

>gnl|Egra|Contig2023 [NDTB12]

MVIGTFFKTGFEKGLPLHEQVVRHLLPLVPKARKGFWPYYFAVNERVVLPRRAGAALNSRLRIPGKNRRECLPTSASSPLELAQLRKATDKPVEDVKPQVFVSTSSPSDAVPLHNESVHSKWLEALDEVNKTASTFSDAFEIQNESLSKEIFHRLAVPASLKAGNIFAHDGAFGSNSADDIKFTAVTHDPTAALFLRHMVNPVPQVDPVDFPNLFSVFHIHDYEFTDPRIVEEFDGVKKEQLGITSPRFVLYDLAERNVYVSGSSQDLRDAIACLGGLVAFHLYGSLTLACNSFIDKDGKLTLVFGSEANLNSPQLFGAHHSLWTPNGVSRAWNGVTVEGAKAQFASDLVEMTAKGPRLTAPLPLQLGGTARPRGANTAGGGRRQAPRSPPWPWTPSCPGGPTWCRRQGPSSFLLERKRRSCLWTMQQRCFADSHAAYPLGFSTKKKLAAKFKELAATTPGASFVTTPNVSSL

>comp62912_c0_seq6_c [NDUFV1]

MLLRGVRCGALAGKQRRSIIQVPDKAPKTPAGFLQDKDRIFQNIYNDHGQGLKNAQKLGDWYRTKDLLAKGPEWIIDQIKKSGLRGRGGAGFPSGLKWSFMPKKPPADGRPSYVVINADESEPGTCKDREIMRMEPHKLVEGTLIAGFAMRARAAYIYVRGEFYNETLSVEKAIKEAYDAGLLGDNACGSGYKFDIVLCRGAGAYICGEETGLIESLEGKSGKPRLKPPFPANIGLWGCPTIVTNVETVSVSPTILRRGPEWFASFGRKNNSGTKLFCISGHVNTPCTVEESMSIPLRELLEKHCGGVRGGWDNLLAVIPGGSSVPVLPKSICDDVLMDFDALMDEGSALGTAAVIVMDKSTDLIKALARISMFYKNESCNQCTPCREGAGVMANIMDRIWKGNGFGPEVDMIKDIADNVELRSICALGAAAAWPIQGLHKHFREEMIQRIEDFTDISYNLHKDEMFGHGAAFDWRRTPKTFHGTLDSFQDSTYRPPAANNNAYRGQYPHVPIYEKEKSEADKIAA

>comp62122_c0_seq3_c [CAG1]

MAKLTWLKNLLNPYSRVPPSTFLRFFPRLLNKFSSAERDINAEFPGTVHKHIKTYQERFMEQGAGDRIATKWNPKPWEKAYMGQPDHPMTKAEQAKKEDFMVGIHWDRSAGGRWTPNDKFPLFDYEFPIHPGRIILRWLYKQGKEPVNMQRSILVTDDFATPSVYPFGWHAPSAILIGDACISNDAAVFDHCVLRADRAAIWVGPKSHVLEGCTLTTAPPTPDRPALGSVLIGENTVVGAGSSLNACWIGDHCIIGSGCTIGFGARIDDGAVVGAGSVVEDDQYIPAGEVWVGRPARYLRKTGDVDTFTAVAENDTLRSLHLAYSEYETTHGNVWAESDKVCDNLEEEVAHRLQAHDVARAMVSKNFDAKLLKLPKSLVADLMDIVSDDDHPNPKPTVSAQARQHFSSQWDFNRKQEQRPVFTGNYNSPTMSRDMA

>comp63840_c0_seq1 [NDTB17]

MRRVGVYALRCARGHASSALAAAPASETLTVGSLYSKKLREYPHKVLWRRKTGFENSSWNQCRRSHEALATNFSEIEVGDRALFITDCDSLMVNVALAIQKRGAQVCIVPAQGLTSNKLETYIEQLRPKVIFLGKDKVSVPDPLGGDEAPQKMTLDLYSMIWKIFPISNVVEGLPLQSKRYPFVKSVVLCSQVDAINQHDMITNIKYFETPRDQDYYESPLVHIAMHIAPDFPALSVVDSAGKWTTHSHTTLLNAGRLFAAKYFGKAEHVLLLPGSESTPGGILTLYAALHAHAVLCYGEDQLVTKGDCKRVTRAVHQHEATAVVGTKAQWDFLLKYGDAATKKEDATKEWFKSLKWAAVFLNDVEDAGDLASKIRSSFGIDRVECIRGSPETLNLTSDGSGKLFDSLSVVIKDKDGVATRGDAEGFVWIKGPHVSAQYWNHIGLMSAPKDPNGYVKLPYVGKKKPDGGFTITTVQPVRELVL

>comp61975_c0_seq5 [NDUFA6]

MPQMRVATKGVRPFSVRAAGGQYVLPDHGRYGQVVRPARLEEFELNPHQNPSRDRDWSVEIRGFYRDLLKSIPTMKQRFRLVIPNDVVRQNIRKRFEQGPKLTDPAALRHRALMVSADLEEYFREDFLDSQVQGKYNNMDPRTLLNQEIAAAASETQTAHRFFNEGTNVLLETGIGGEDVTENRVYITREQAYRKGLASLRGDAAVRHLLPAVDPANQTTLQALAAENDLQALVDLLGHLPAAKTAEAYVQRCEAFHKEAGLRHQKASGGAVLAAWEKFKDEEVNSTVLLHPAYKALIADPSRNPLLRGAADWVRLVEAGGLSTTEPDSAADKLLKVAQHLYYSDQLPEGFAQDLGVSYLADLKGVDRRLDLLLDEEIAYRQELLLKIYAHTVESIKATASNPTDPAAVKKHLDAHDWSAFVVPTEGVKSSYEALAL

>comp46408_c0_seq1 [unknown]

ADLKGVDRRLDLLLDEEIAYRQELLLKIHAHTVESMKATLSGPADAASVRKHLDAYDWSQFVVPAEGAKGSYEALAL

>comp63125_c0_seq1 [NDTB2]

MSRNVLTKGWRFHAHAGTFQSALRFEEFQLKKSVGDVVLRLQCAPVTGFDLDQVRGLQGKVPLPAVGGLSGVGVVTEGSGIFKEGDRAVLLGANGAWSQYAVSSANHLLSVPATIPVEYASLLASGPFAAYRILKAAHLKAGDLVLVNGAHTAIGLAALQIAKAWGIDAVGVAHGAPALQVEKLKQMGLNVVSSFALDPKQVFGTSQPKFAISLVGGNAAAYVTHLIGSDGHIITCPLASDEPHILPNVDLVNKNLTIQTFSPWKSLLSATATENEQMVSELCDLIAAHKLKANAVVRHEFGNLLDAIREAEHGTHNAVILHEGTEKTWDNKNHDIYMEIDDKLQANWDAAAAAQDPYLKTGRDQPWQVLAEAEEVALPDELRVKLAAVTTEAELLAVLDTLTLKERHLLGLPATQAITVSAEELKKMVSEFAS

>comp55715_c0_seq3_cut [NDUFS2]

MMMRLTKNLPRLGIATLQNRFFRANALKQMPARADARTMQHFTLNFGPAHPAAHGVLRLILEMNHEYIIRADPHIGFLHRGTEKLMEMKPVIQVTPYMDRLDYMSAMANEHAFIMAVEKLCEIPVPLRGSYIRVIFLEITRLLNHLLAVACAAADVGALTPILWTFEEREKLMTFYECVSGSRMHPNFFRPGGVASDLPYGFLDQLYEFINQFAARLDEVEDLLTQNNIWRERLKAGYMDLDRALSYGFSGPMLRATGVAWDLRVTAPYEVYPYLDVEVPYGVNSDSYDRYLIRMQEMRNSIRIIHQCINDIVPGDYRLHDAKFATIPFRDCKDSMENLIAHFKYYSEGFRIPEGFVYCGTESPKGELGVYLQSNGDSLPYRVRIRAPGFYHLAALPALAQDTMFPDLVTVIGTLDIVFGEVDR

>comp60945_c0_seq5_cut [DnaJ]

MWRTSGCALRSRASFTTAVTAHASGVKAIDPFKILSLPDSATRDDLRNQFFELAKSNHPDVGGDKAKFQAIQDAYEDAIRIADQKHPVAPWDGISPMTYAQAWQGKDYWRKLWEEHWAARLAHMYKHNAELTTLEANKKWREAQYMQVKDWMVLAKDVLDPKTKAEWQAGCELARDMLLWTQANKKNYRRYFLSNQNVAVNMRQVYDEHEYWRQYENVQWAQWDAFFARASAWALEHEEQIRSVNSTEGPLAAKFDYLFHGRLQYSSMSLEERLSRRAQEEKAYTRQYWIAELMKAMRFSFRWVERFSRAFFPVLILVVIAGYITDFQLIIRWLNITRSETGALEVHNRKMDMVDWLLAGTPTPQNIEGTI

>comp61469_c0_seq1_cut [NDUFS1]

MRRVLARFGPHVPRSFHTTVSRLQEAVPASILNAPVGLQPSQTVTCWIDHILCEFQYPADITVFELARRNGINIPHFCYNRNLPIAGNCRMCMCHRVSDKKYAIACNEIAEPNAKYITVDDNLKNIRQYILEFILANHSLDCPICDQGGECDLQDLAELYGYDTSRYDYSDIKHEPDDMPINFLIKSDMNRCIHCTKCVRFLDNFSDDGKEGELGLMGRDPQTICVFRDDGNPQSYVADILSANVIEICPVGALTGRETNHETRPWEITRLDAINIFDGTLSAINVEVKEGTELYRVNASKDPQNPDMLLNNEFITDRAREAPQGNEFKRMTANYAISLDNKKLLLHHALRLYAIDPLFRSKALFLLADIMNEDRHLPETKQLGH

>comp59654_c0_seq4 [NDUFA9]

MQSLKRAGTLSRPFLRCFYDAGAPQIFRSNVPGRPLPWRQERQVPPNPSQSKWQWEPEHIPTAEEYEAFPEVITLYGGDGLLRSSVIQELVQSPRVSTIRVGTPWPDEFASKLPGEWQSKVVAEFVDILDRHSVLAAAEGSQALVNMMDIPYECELTYYQAHVGSAQMISHAANTCMCSRVIHVSSLASRVDSWSRYSESKFRGEDMSLACFPWTTILRFGPLVGKNSPALKQFASYMKYAPIYPCVAKDTKIQPTFVGDAAKAILAALGNPSTRQLQFDLGGPEVFKHADFIKEVMRLTKASRPVVPVPGVIGDSIVALLQWLPDPLVTRDMVYLIRSHHIANHDSMRTWKDLLPEHKLKTMAEALQ

>gnl|Egra|Contig149 [NDUFA12]

RPLFFAMIRTLLLRHSAPAVITGRPQWWTQAIAVPPTQAEMELFQPKEVVHTKPYKPHPWFKDFGQGRRHIVGPPERGEFWRFRKFYAVMREKTKELGVRGALRFLVRKLRTQREAWYEKGYEEDILVGEDEMGNKYWQSSYTTAVQSRWVEYGTGQHFHEGRQRRGAGVVPMAARGPRP

GSPGASPKAPSSFDQRLDW

>comp62013_c0_seq2_cut_gi|109788168 [ADP/ATP] MAEGKKKGGVAQLVQDLFIGGSIGAVSKTVMAPVERIKLLLQTMDSNPDIRSGKVQPYKGIGDCFSRVYAEQGPLAFWRGNLVNCLRYAPQQGSALAFNDMLNNMFPKYDSRTQFWQSLGSKLFSGGLAGAIANTICYPFDFARTRLASDVGSGKGKFNGIGDCIMTTVRQQGITGLYTGWSVTVAGAFVYRAGQLGCFKQIQDLNPYKNDKGTLGAVSAFVAVTAARTVVMPFNRSEERVRRRMMLQSEKPIEERLY

>comp52123_c0_seq2_ gi|125990644 [G3PD] MTTIHAYTATQKTVDGPSKKDWRGGRAAAITMVFRPLFRSFVNPFSLARLMAFRPTSVLALRTCQLRLGGGHGHGHGHGHGHGGHGGKPNKFENHIYRDASGQPWFDLHKWELGRKPGKEDLEHPGRHESFSQTKRLVSLTFIDKDGNPHNIKCEPGMNMADAALQAGVSLDWLDGEGNQGFSAWSHMVISSPQFEMLVPPDHREDKYLWDLEQWGTSHRNSRFVKFLFVQPWMEGMVVTHPFFADVVTPNFTDPFTGKIEDKDQAVAPTYFVSKVVDPEKPDYKFPNPFELLWAGIDTYEQLWDYTRQSWLKRLEARRQKQQQA

>comp58177_c0_seq3 [unknown]

MFQPVWQPILMVGSPDIILHSAERRALAWDHPNRFSALRNALYQARLLEQPRPENRIALLGQDLLEDTIYTTVGAYLFAGVSCIQRLGGHVPFTPSFTGQNIWTMPKWASRLLHQVRMMRYFSAYWAVGMTYFTTYNILTGFMGFPVNEYHNYQPQASVLSVIPTALIYAALHPNRRPERLWVGKATPFVGRFFLSGIVGAALAVFAARRFAHATVSELYHPSGSDSYFETLRNSAPSADLVADMPYIPFYKEARCSPGLPVKSPYYDPEYVAKAKEEVKRKLDSLY

>comp62960_c0_seq11 [NDUFS3]

MFRRVATHVVPKLSTLLRARNSIKFVTTQEVPPVHQGREVKSTPQNKGKDEFVRYLSSVLYPMASEIMWDDVTNDVTINVYPQYIRPVAKMLRDHQQFQYKVLVDVTCVDTMTNLKRFDVVYNFLSVVFRHRVMVRASVDDASGIDSLTPLFHSAMWAEREAWDMFGVFFVGHPDLRRMLSDYGFEGHPLRKDFPLGGYTEVYWDAPNSTIKYASPPIFREEYRDYENIETEWDDLYHRCNTAWAYDRMVKQGMESCDADYTYDDPAAHKKPQQTQG

>comp48089_c0_seq3_cut [CAG2]

MPLPQKSTLVRLGAAIRECGQALDRWGSFLQGRYGHLEKLQRTRRINGFHNFFPEVKGVRFIAPSASVIGQVTVSPGSSIWYNSVVRGDRGKVTIGEDTHILERVVIRSGILSVRDVKIGKDVIIEPGAIISPCQIEDGAYIGANAVLMEGCKIGKGVVVGPGAVVTEFAELTQPGVYQGVPAKSATALTTEAAEAITTRRAEFAKLAEEHEEMNTKLIEKQTEERVILKDILENQLNEGNEFTMRSHHVARAPNVSPGNIAAGSAA

>comp47716_c0_seq2_cut [unknown]

MTTHRPPLVTDRMFPWTWNSRYYMMTPLATYGREDIFANPHLPADKKLHAAAVEGGEFMKDAYAPPHKAVSLVRRVGRKAMVGAGLYFHLFLMDKFVSKGLLDRNTSPYFYMTLYEKAIYCLLPNKNKPNEEDLLKLFREARLESNYKSVSMWTYRLRSERVPKSYFDKPPVSYDADRRTSGFVEWAFWMVGLESFGKSVVPEAW

>comp51732_c0_seq5_ gi|125990596 [unknown] MIRALLRAAPAGLAARPLHTSAPRLIFPFRRPFKSEPEDETKIPDCYVNGKPIADGQGYQAIGLAEHFERIRRNVNIRRRVPSRFTQMVGGRRARTFSIHFERGSVEREWNQGGRLGVEPFAMMRRKMKNFPTVEDAEEIGRAAGWKINSSNPGNMRLNPRVAGNRREWKQRYEFAKDSYAAKDSGKGALYPQDPHSSG

>comp59406_c0_seq3 [NDUFA13]

MAEKAPEVHHPQDKLPEWFDPNSQQFTRNWRQAMPPKGGYPAVRHRYLSPGKGVSMRTAWAIIGSLTLFGLYQRRVDRAFILDLGDDFHARNCAAVPYMEAENQLKDLISLHYEKEYRNFVAPGLKLDPSQFFNHPIPGAVGEYIRKTKPQRSTKNRVAWMNLELCTEPISQLSGAPQSGPDKYERMLGFTVYEGPAF

>comp52747_c0_seq1_cut [unknown]

MDRSSVTPRQVFLEPVELPFAKKDASLEIFTESQLKSLLEPADASLFKWKNLAKWKKEVRTLLPQEFQSWDPISNQETIVDLNPYLVGGIFGGFNAMAHNYLSCTVWWKVPWRFPMWMAAWAGVSAYLFEFRREVEYENRKSRLLVNNYYNRVRTVIVEREKRKNGPDHQFKDLPWMDREEQRSG

>comp54309_c0_seq4_cut [NDUFS8]

MQHLKLARRGLPLQQLRSFHWTAPPLLRQPHPDVPKDVSGLGMGLAFMNQFLLKEIMKAMWIVTIYTFRQPSVTIHYPYERNTKSTRFRGEHALLIYPDGDERCITCQLCEVTCPAQAIAIDGVEDDDGSRMASRFDLDMHKCIYCGLCQEACPVDAIVETPHAEFCSTEYDSLLYDKARLLENGIRWTSAIEYMVAKERSDFPTNSQRFKS

>comp49716_c0_seq2 [NDUFA5]

MFRAALRQASSRAAVCRYATKVKDFTGLAGYPVEPKWRGKLLALYAETLEAVQGIPAGHPYRRSVENLTRHYARVVEEAEDYEVVETTVGLGQVEQLIRMATNELQLVRDFTVWRTWEIDERVLDKMDVDLRTSIGGHYTKRAYEVLDEVEDERRLLEKLQQEVDLEKARVAAKAAEGPKTL

>comp49825_c0_seq3_cut [unknown]

MAERTFRSLGRLPAPSAPLPHDKIAWPEKIRFLPEGSIMMPDGYSLDPNQHFEDPYHWSVLRRAWEPKQDVFVGKDSHGNVVKQYNSPQVRHLHPGFKLFYLYRKGAARRFQVNAQNIAYAAIFAGTCLALCEFGLRVQNWAGGVNTLEKLQSTERKVKMNGKVIVAPDSF

>comp56597_c0_seq3 [p18]

MQKLSRVVCNRLVRFHGTVAASAGGKRYDLFGYEVSVATGPFIEEIKKAQFYDDAGEVIVKMNLANTPPDLQTYNAVLERILNCKSKRSQPVKGENKFAAMMDILEEMDARSGIKPNAESWGYVLKELVQAGDFRLGWVCIAGMKSLGITPDQALVDANEANAAKAKAAGTDFPAYLKKAAPESFDTKAWGI

>comp53986_c0_seq1_cut [unknown]

MKRSLLKLKDLKKVKVTQPQSSIVLKPVPIGEPIIWSRVQHLYWRALHVIEDFPWMGPSQTSEQFDIMLSVKEEIQKNANAAGALKDRLLEDFEAHIKRRDWMQRRVHYFANGGPGYQTFASERHTLEQQGVWKAGTPIPFPKSDYNDDGPVEKGPYY

>comp55416_c0_seq4 [NDUFS6]

MATTSRLSKVTVNIMPASRALGVKPHRHRKQWQMPEVSFLKDVFSDPLMTEQKITERFIKAEFGPNRIDVRRAVPPIVVPKTFKHAWCDGNWGNRSTHGHPRISIRMKAGKVSQCKYCENKFVRDDFPAFFAKREQVAAEEKYHDED

>comp41364_c0_seq3 [unknown]

MAHDKPHNEPPKRLLSKQEKTDMAVRENIEKPSWIKENGFQRWCYEMVRGVGRHPWPRYFELTDFFGDKRHGDAIPRSWAAVKKLERGQVIGPAVCVTLTLWVFVAGTISKRNNTVWCDSIQEKYGFKQPYSREFRTQHDGVLKPKLPAIY

>comp54566_c0_seq2 [NDUFB10]

MGQHFGKLGWDADDVYKVTTFEKHNYTVEEPRTPAFKAPRRPFPICDRPPMPDQEGLYETHADLYEMKRWVMWEWSVYCKELSLVREELKWCVAREGSNAHRECHDLARQYTDMTRKLKMEERFSWLNHFKKPLVKDVLFDLKQ

>comp54442_c0_seq1 [unknown] RSSDLVRRGALIASLVRNRTIRPKPLQVRTGSGGHHSHSVDPNDPFDGVENELFLPPQAYYYFSRILFVTTLIVWLKTYRDKAIDQFSFKKWVDSLPEMTIKEEEIYLDEWEDWEAPQHDVARMFPRYTKKFRKADDWNFGATVW

>comp54200_c0_seq1 [unknown]

MPGGGGWSNMVPIIILNGVVWAALGRASLACSPPEFHKRTKNDTEFNKYLHLRFNKAVQNPESVAGQAVKAGCAPEFRPFDSPANPLVVVYGWKDEIQPRPNPGSLAQSFDDRGLSWYQSHFSNRVVDDPKHNSLPFPGYY

>comp51611_c0_seq1_cut [unknown] MDRYEFQKIRRQPPTLHWEAGNRFENIQRLRWENAALLKDPKLTWFRREMLMRPAFFHCTLFAGAVAVGYPFVAYFYEKVFPDRQDFRSTMTLLRAVGGLEEQEYYIMERAKAIERAKARAAVQG

>comp51117_c0_seq1 [unknown]

ALPIFFSQMDVHSLSRLMVPLARRSPLLLRGGVSRPVRSKRETDFNSSDPWETPFGRIEHDDEDIPYTTTVYQPRRPTEWSTDRK

**Sequences Complex III**

>comp63646_c0_seq8_cut [QCR1]

MTTPSLSSILRHTRPIFKETLRAARPTLQNALPNGFRIASESKDGDTCTVGVWIDAGSRWETEKNNGVAHFLEHMNFKGTGKRSRQDIEFGMEKMGAHLNAYTSREHTCYYVKCFKKDVPEAVDILADILLNSKRTEQDLDAERQTIVQEKEDVEARIDEVLMDHLHSAAFEGSGLGLSILGPLENIQKSITKGMIDDFVKTHYTGPRMALVGSGAVDHGQLCDLASKYFGALPTGQPKPSGFTRFLGGDKRETNQLNPLTHVAVAFQTPGISHPDAIKIKVLEQLLGSYSRDKGEAAYSCFARAIVMDFYDPKVGQFFRPNKAGHNPIHSLNAFWAPYSDVGLLGFYAIAEPGKSYGHEWENILHYAMRELIRVSRNISEEEFERAKNQLKLQTMLQLDGTTNIADDIGRQVLSFGARVPLASFFEQLDAISREDLIRVAHEYFYDKDPVVAVIGDTDNVPEYDALRAVTYSVEIGRA

>comp60854_c0_seq1_cut [QCRTB1]

MKSVVRSKGTQALFRRFSSALGDSINPNQVGVGDNVIRVNGRLFEVDKVQEKGLKTSVLDNGTKVITLDNGGSVAQLTFLYKDGPVYENIFNAGISSFMKHALTKDGLTSSEYITKTFLQKAGIIVHEPTVVNKSAIAFTVEGFRDTLAQPAVADKFWQSLLFPRFSPENVKEVKRLVELESKETKRDSPFAYLQDILHKTAFKGSPLGHTSFVPAYNLGYIDSNKLFDRWDAHYGFGNIAVVATNIEHEAVLAAITDSAWVARAHNKVGGVAAPASKYSGGEGYDVVHRAKEFDDQFTDVYSTYTAYAFKAPGRSNLKEHAASLVIAQALSNAVSPVLNTSFAPKRLEVFYQAYDTVGLIGLSSVQASNAQLKAFKAALSKIGTLSEADLAVHKSAALLTAYGNVESWRATQATLIDSFNTTGQPLSPLEIVSAIKAVSADTVKSVVATMLGSPATLVHHGDSPCAPTLDALQ

>comp49373_c0_seq3_cut [CYT1]

MGVDSHPPALPWPHFQWFQGLDWRSVRRGKEVYEQVFAPCHSLSFIKYRHFEAFMSKEEVKNMAASFEVDDDPDEKGEARKRPGKRFDTVVQPYKNEQEARYANNGALPPDLSVITNARHGGVDYIYALLTGYGRPVPGGVQLSTTQWYNPYFHGGIIGMPPPLTDDMIEYEDGTPASVPQMAKDVTCFLEWCSNPWWDERKLLGYKTIATLAVIAVSSGYYNRFLSGLWRSRRLAFRPFNYSK

>comp57718_c0_seq1 [COB]
MRELDTGWLIRFNHINGCAFLFIVIYMHIYRSLYHNSITKTSVWIVGIIMYILICGIAFTGYSLVYGQMSLWAIVVICSLVTAIPFIGNKLLILIWGGNIVSSVTLQRRSE

>comp51517_c0_seq3_cut [QCR7]

MNDAPILAKLGLSKVNTYYFQRRKHIFALPITIPNFPLAALGIFRSTDMFEERTLERYSRDEREKTLGKAALLLKEAKEKGNYSELVRLDPTDPRHPYYFEHPWQSALKMDTVSLTPYQQYLRWHCLTYRAMHEWDRQGLLYDDLMQPKALATDPFLEEAILRLPYNQRVERERRLSRAYDLALRREYLPDEDCIHPEDDVAYLHPYYHMVVDEHREQHENPVDVYSR

>comp57996_c0_seq3_cut [RIP1]

MFKQLSRMYSTSPAVANAVGGLATSQIRDLVGNPAKSGKPVVKMPPYLKPSQLRPSRGAYSDEFVRENYIKAADPEDPYNYRSRAFTYAAKIPMWAGLLAGTRVTVVYLMSQFMPSKSSLALANIEVDIGDIPEGKTVTIMWQGKPVFLRKRTDAEIEDMRAVPMDALKDPQTDEERLGEGRWAVFMAVCTHLGCVPVIDQGSYNAYFCPCHGSHYDHSGRIRKGPAPLNLEVPPFKLLDDSTLFIGNADAA

>comp47102_c0_seq2_cut [unknown]

MLSRLLHGPGSDKGHGYFHPDHGLVDSAAQNIRGPYWHAENMQFMQQTTQSGEKLPVPLTETAAQSSKLPVLSAQDGKTLQKIQLTDGSKVPKAATLVKWNPKSVHQWHSDDILRVDMTKHPEWVRTMRMFHGTVWRHRPEFRHPWFSRGSRASGIIMLVFGAVAYGEITFGKRLAEFF

>comp57617_c0_seq1_cut [unknown]

MALEIKREWHVVPATKKFADVPTEWPAKEHERFALPYGPSEGRIVSFMRASRIRNRLLFTLNPTGYTEDTYVTKEALRRQALAESKNLWYLPERPVVADQLETIVNRSTFFVVKGHKNKGLIGIAGARHNPLVWLPTLALGGVWAWRWASAGTI

**Sequences Complex IV**

>comp60817_c0_seq2_cut [COX1]

MHMINKYTLTTSHKIIGILYGYMGYIAGILGYIISMLIRMELNTQGLAIVRKVKEVTIYNNWITIHGLIMLFVFIMPVGIGFYGNYLIPMLIGTSELSMPRMNGISFWMLIVGVVIFVISNVLMSKPISSGWTLYPPLSTRDADNIGVNIDLSLLVVHVLGISSTIGSVNYITTNKYNRHVGLTFMNINIYNFSIIVTSILLIGSLPILGVAITGLLLDRNINSTIYDVIGDPVLYQHLFWFFGHPEVYVIILPVFGLTSLILTSIIHKDIFGREGMMYCIISIGVVGYFVWAHHMFTVGLDIDSRSYFSIATSIISIPTSVKMFSYINTWASGRGFRGNNSSWSFFSFLICFCFGGFTGLLLSSGSLDIMLHDTYFVVGHFHTVLSLAATFGLLIAHYFRSEERF

>comp55436_c0_seq3_cut [COXTB4]

MLRQVVRRSNPLRMQVRGSAWNFQELMESRIPDYKGRPNRSGAELEQVKAALPKIEFMTSYEFDVLTKTRSNLTKEYSYQRDMRLKVTELMLDEAPHELEGLAVEGDAAVKQLAELKALQTLTEYAGDLLEGQNQIVQRVNDFVDSNPVYLLDQPLREEARWNLLPEMDHKTRSLVRTELRDWLPAEYRQTRAVDLQQVAAFSPQVKADMFRAIEARAKDAEAEIRSLPPAEQAGLLALVKDNVAKSKAFIDPTYDITPEAINACNDVDALRAMAHRVTEYSGDARLLAIYGKAAQLTGDTAAQAILKEAKDLVF

>comp54364_c0_seq4 [COX6B]

MEGFVDKIDDNKYLGKWETILTDGRTHLPKHITFHDAAAISARWNQQYVNDSGPVYYRHWLACQQTYGAGNEDCRKLRWWAQQITHPLHLAEWDDWWKDEHYDLQIGQHWNRICGEEFEEASNLLKDLKEKREGLAAKFRDLLKTKTAEDPMGKILHEVAQLEEPSKTPVADLVEAGTLSKEAVEAAAALKIKELKALRDDATWAEVKGSLLNGVTTTCSTLKKTSKVVAELKAQAELERNKTSAVKLDIPHMRVNYEKPGLYEYDTWFGKFLPRTPQFGFAESDEE

>comp57506_c0_seq2_cut [unknown]

MMNKGRILLGTNPGDIALNSKRFTVGKFVAWACGGWGLKDWIFPSLFIGRGDGPDFDRIVKHTLQSSSAIEKVNWFDSPFACYTEWFVEHFPGFFDSRYRFEMSAKTILANKYPIKDFPVVDMRSWRSSRLFDLFEVPHPEHTFVFGGPVLLNTEAKRAERLEQEWHGKDGTFVDVHPLNVATESHTEVSVIGGIKVYNGVWQGGKDSWKRDSAKPELTAPFHSPIWYRNMFIVKNADQLVEHFGENLSDETWQEVRKEHLAFHERFHKDYSFA

>gi|109781798_cut [COXTB5] MLRVLTPAIARPGLRCFFKDGFRDNASLELVYRVVLKSPAVSQKLIEFYAKSLDQLSVESLSALKGTTVGIPLQPYLGDPHRVLLAYSLLPHTVETEADGNPVVETKIGDEEQKIKIIDSEVISFLAKEILGKLGLETTPQAARQYLDSLVEGAEALYAKIAPVEPSPLEKAIAEINEEIKSGTPWDTLKNRADPKELHALKFAQLPHPITKKVEGKFKYF

>comp54737_c0_seq2 [COX3]

MLSRALCSRNVPMSLKALNRPGSAGKLPMQLMKYASAVAPISHEGTLVRISQVKKLSELQLHFNDSHLGESELAAKVLGKLRKLEAEVLARNQAFNEAHPLVFDPKRAFNDEIFLCCSLCCIIFLIFLFNQYEEFAHELSFDIREQFGLGFYMLLGLHGSHVIFGTIMLALLTLWGAQGSVGPQSHALRFTSLYVHLVDLVFIILVLAIYSANASPELYGGIVPNILEARTFVSVDAAGNPQIKEF

>comp53543_c0_seq2 [unknown]

MGGDAHAHGGHGLHADPIFNGSKVVRSIQMMRSYHRLPVGPEPPHLKIRGAPLHPWSMYTDKGGFFFGVGTQLPRNFFPKFLATTSAIMGVTYGLVWLYNAAGPRAKTQTRKWKELESEDPRFPFQEIPEIYLPSDIDRDPNADCWRILNQVPRKEKLLVEITDPLRNFDFKVAKIKEN

>comp51338_c0_seq1_cut [unknown]

MPSSMAWTIGWGFYAAWIMKETWNLRSSSVGWTPITLMEAYKTKERYLRSKAMMERYNSELEAVDDSNITEEDAAKFELSKATPSISIWEQFRSNPYWKEVEEEISTDVRKTMLEKHPDYALLLEAVKKSGYSKLWHLPGPWMNEHYNDGLHGRFLGWTPKAAHQDRKS

>comp47102_c0_seq2_cut [unknown]

MLSRLLHGPGSDKGHGYFHPDHGLVDSAAQNIRGPYWHAENMQFMQQTTQSGEKLPVPLTETAAQSSKLPVLSAQDGKTLQKIQLTDGSKVPKAATLVKWNPKSVHQWHSDDILRVDMTKHPEWVRTMRMFHGTVWRHRPEFRHPWFSRGSRASGIIMLVFGAVAYGEITFGKRLAEFF

>comp54722_c0_seq3_cut [COXTB2]

MFRRGLVLAASRSKSLLDSVHVFRPEFAQGKFRIDLDQPAKQNKLQQQIYTLTDDEREMYEDEPYIGVDHLYEAHKGSKENPVVVEAIGVHGNDVFTGCLGGCHKDAADAVAYYTIVPPNTLAVCIDCGIHFVARINEQLTFWPDGTQPWEKVDFKAVEGFLYKHYKYGSPLMI

>comp55710_c0_seq1_cut [unknown]

MQKAVVGNLLRQLSLTRPRGHGSNLYNRVHGNLPQLYVEQLYNTDEILDTVPHSSDPVHHMFPKCAAASPLGFRPYDNNKLWDAFVLWAIVYGVTTIFCVLHILKYPQIWKHLFETLTFQYTYQREIGEEYVWQYGGGGLNPAKWRFVKSPLEELGLVTEYRSDLDYPDDL

>comp50120_c1_seq1_cut [unknown]

MMQRIPFKKPNQIRGYFTRVHKYNHVPVPFILNVGMSISIVTSFVYFTYTSLWVRPEYDRVVDPSKAYVNPVWVDYWLKLRDEKRIQGALERSILEEEPEKAAEKILEWARTSAQNKILEDLKLLKPALSPATIAQFEK

>comp48005_c0_seq1_cut [unknown]

MSTNKNVFFPPALHLKENSIFQYKFKNLALRHDAARLGIILAGPTLFYWFTVYYFKGMPNGLPPVLLNPFVNRNYRGQKRDMPWGTDCAFLDTKCHEEKDTWAKRGPFFIIA

>comp53374_c0_seq4 [unknown]

MNHERPWVFLNKVTGKWGACGWQPFWKATSQSLNYIPDNFIALRTNGSWVRNLWQSSKVLDRGLNASGYPTVGTTDWVRIPQIGR

References

1. Zickermann, V. *et al.* Mechanistic insight from the crystal structure of mitochondrial complex I. *Science (80-. ).* **347,** 44–49 (2015).

2. Shimada, S. *et al.* Complex structure of cytochrome *c* –cytochrome *c*  oxidase reveals a novel protein–protein interaction mode. *EMBO J.* **36,** 291–300 (2017).

3. Atteia, A. *et al.* Bifunctional aldehyde/alcohol dehydrogenase (ADHE) in chlorophyte algal mitochondria. *Plant Mol. Biol.* **53,** 175–188 (2003).

4. Cardol, P., Figueroa, F., Remacle, C., Franzén, L.-G. & González-Halphen, D. in *The Chlamydomonas Sourcebook* 469–502 (2009). doi:10.1016/B978-0-12-370873-1.00021-6

5. Miranda-Astudillo, H. *et al.* Oxidative phosphorylation supercomplexes and respirasome reconstitution of the colorless alga Polytomella sp. *Biochim. Biophys. Acta - Bioenerg.* **1859,** 434–444 (2018).

6. van Lis, R., Mendoza-Hernández, G., Groth, G. & Atteia, A. New insights into the unique structure of the F0F1-ATP synthase from the chlamydomonad algae Polytomella sp. and Chlamydomonas reinhardtii. *Plant Physiol.* **144,** 1190–9 (2007).

7. van Lis, R., González-Halphen, D. & Atteia, A. Divergence of the mitochondrial electron transport chains from the green alga Chlamydomonas reinhardtii and its colorless close relative Polytomella sp. *Biochim. Biophys. Acta - Bioenerg.* **1708,** 23–34 (2005).

8. van Lis, R., Atteia, A., Mendoza-hernández, G. & González-halphen, D. Identification of Novel Mitochondrial Protein Components of Chlamydomonas reinhardtii . A Proteomic Approach. *Plant Physiol.* **132,** 318–330 (2003).
